# Correction: A neural network model of mathematics anxiety: The role of attention

**DOI:** 10.1371/journal.pone.0331062

**Published:** 2025-08-26

**Authors:** The PLOS One Staff

## Notice of republication

This article was republished on August 11, 2025, to correct errors in the Data Availability statement. The publisher apologizes for the errors. Please download this article again to view the correct version. The originally published, uncorrected article and the republished, corrected articles are provided here for reference.

## Supporting information

S1 File Originally published, uncorrected article. (PDF)

S2 File Republished, corrected article. (PDF)
