# Supplementary material for: A neural network model of mathematics anxiety: The role of attention
Source: PLoS One. 2023 Dec 14;18(12):e0295264. doi: 10.1371/journal.pone.0295264 (PMC10721013; doi:10.1371/journal.pone.0295264)
Supplement: S1 File — (PDF) [file pone.0295264.s001.pdf]

S1 Model architecture: Further details and equations

Single-digit comparison module

The equation for the activation of node j when input number i is presented to the model is as per Huber et al. [1] (and is analogous to Santens and Verguts [2] who used an exponent of -1 instead of -10) as follows:

$$f(i, j) = exp(-10 * |i - j|) \text{ where } 1 \leq i \leq 9; 1 \leq j \leq 9. \quad (1)$$

The numerical magnitude representation reflects place-coding properties as each input number presented to the model activates the same number of units on the number line. Each number presented to the model demonstrates constant variability by maximally activating its corresponding number line node with adjacent nodes being activated with decreasing strength as they become further away. The model exhibits the properties of linear scaling as the exponent -10 * $|i – j|$ relies on the distance between the number nodes and not on the actual value of the corresponding numbers i and j.

Propagation of input to comparison layer

Activity is propagated similar to Equation (1) of Moeller et al. [3]:

$$\overline{net_i}(t) = \tau\, net_i(t) + (1 - \tau)\overline{net_i}(t - 1). \quad (2)$$

where $\overline{net_i}(t)$ is a weighted sum of inputs across time t for node i, τ is a constant of value 0.01 reflecting the rate of activation, and $net_i(t)$ represents the activation of place-coding

nodes multiplied by the connection weights between the input and comparison nodes. The net input activation is then transferred by a sigmoid function with a gain of value 2. Lateral inhibitory connections between the left and right comparison nodes with w^{inh} = -2 create competition between the nodes thereby strengthening the node with the largest amount of activation and weakening the node with the smallest activation. The activation $f_i(t)$ of comparison layer node *i* is calculated as follows:

$$f_i(t) = \frac{1}{1+ e^{-2(\overline{net_i}(t)+w^{inh}\sum_{j\neq i}\overline{net_j}(t))}}. \qquad (3)$$

Training of weights between input and comparison layers

Initial weights were random numbers generated from a uniform distribution in the interval U(-1,1). Training was performed using the delta rule [4] with a learning rate of 0.01. Tuning the learning parameters to increase performance was outside the scope of Huber et al.'s [1] study whose objective was to create an abstract model to capture multi-symbol number comparison instead of creating a biologically plausible neural network model. Similarly, performance tuning of the model is outside the scope of the present study as the aim was to create a model that simulates cognitive mechanisms related to mathematics anxiety and not a biological plausible model.

Response layer

The activation x_j^{res} of response layer node *j* at time *t* is the same as equation (2) of Huber et al. [1] with the exception that no noise is added to the formula. The equation is identical to equation (A2) of Verguts and Notebaert [5] as follows:

$$x_j^{res}(t + 1) = (1 - \tau)\, x_j^{res}(t) + \tau \left\{\sum_i w_i^{ir}\, x_i^{in}(t) \left[C + \sum_{k=1}^{n_{task}} w_{ki}^{ti} x_k^{td}(n_{trial})\right] + w^{inh} \sum_{k \neq j} x_k^{res}(t)\right\}. \quad (4)$$

where w_i^{ir} are the bottom-up connection weights between the comparison and response layers and x_i^{in} is the activation in the comparison layer for node *i*. The top-down attentional weighting of the task demand layer to the comparison layer is indicated by the term $\left[C + \sum_{k=1}^{n_{task}} w_{ki}^{ti} x_k^{td}(n_{trial})\right]$, where w_{ki}^{ti} are the connection weights between the task demand layer for node *k* and the comparison layer for node *i*, $x_k^{td}(n_{trial})$ is the activation of task demand nodes for trial n_{trial}, n_{task}= 2 for the two nodes in the task demand layer one for each of the tasks of comparing numerical size and physical size, and *C* is a constant with value 0.7 that ensures irrelevant digits always contribute to the activation in the response layer regardless of the attentional bias in the task demand layer [1,5]. The term $w^{inh} \sum_{k \neq j} x_k^{res}(t)$ represents lateral inhibition between the response nodes.

Cognitive control module

The activation x_i^{in} of node *i* in the comparison layer at time *t* is calculated by equation (1) of Huber et al. [1] with the exception that no noise was added to the calculation (as in Santens and Verguts [2]) as follows:

$$x_i^{in}(t + 1) = (1 - \tau)\, x_i^{in}(t) + \tau\, (f_i(t) + \beta_{in}). \quad (5)$$

Huber et al. [1] adapted the equation from equation (A1) of Verguts and Notebaert [5] whereby the output of the single-digit comparison networks serves as input to the cognitive control network The values of the constants τ = 0.25 and β_{in} = 0.2 are the same as in Huber et

al. [1] and $f_i(t)$ is the activation of the comparison nodes from the single-digit comparison module.

Connection weights between comparison layer and response layer

As in Huber et al. [1] the connection weights between the comparison layer and response layer in the present model were fixed. The values of these weights reflect how automatic the processing route is where the larger the connection weight the more automatic and faster the task is. In the classical Stroop task where the font colour of the word is named while ignoring the meaning of the word, word processing is a more automatic and faster task than naming the font colour [6]. In the numerical Stroop task studies have shown that judging the physical size of the digit is a more automatic task than judging the numerical size of the digit and is therefore processed faster. Szũcs et al. [7] investigated the speed of magnitude processing on numerical size comparison versus physical size comparison. Participants responded faster on a physical task than a numerical task. The ratio of response time from the numerical task to the physical task in the study was equal to 0.94 and this ratio was applied to the connection weights for the numerical and physical size dimensions to the response layer in the present model. Additionally, the values of these weights also affect the size of the size congruity effect and the amount of errors in the model. As in Huber et al. [1], the values of these weights were arbitrarily chosen to ensure the size congruity effect and error rate were similar to empirical studies. The connection weights w^{ir} between the comparison layer and response layer for the numerical size dimension are 0.85 and for the physical size dimension are 0.9.

References

1. Huber S, Nuerk H-C, Willmes K, Moeller K. A General Model Framework for Multisymbol Number Comparison. Psychological Review. 2016;123(6):667–95. doi: 10.1037/rev0000040.
2. Santens S, Verguts T. The size congruity effect: Is bigger always more? Cognition. 2011;118(1):94–110. doi: 10.1016/j.cognition.2010.10.014.
3. Moeller K, Huber S, Nuerk H-C, Willmes K. Two-digit number processing: Holistic, decomposed or hybrid? A computational modelling approach. Psychological Research. 2011;75(4):290–306. doi: 10.1007/s00426-010-0307-2.
4. Widrow B, Hoff ME. Adaptive switching circuits. In: Institute of Radio Engineers, Western Electronic Show and Convention Record, Part 4. 1960. p. 96–104.
5. Verguts T, Notebaert W. Hebbian learning of cognitive control: Dealing with specific and nonspecific adaptation. Psychological Review. 2008;115(2):518–25. doi: 10.1037/0033-295X.115.2.518.
6. Cohen JD, Dunbar K, McClelland JL. On the control of automatic processes: A parallel distributed processing account of the Stroop effect. Psychological Review. 1990;97(3):332–61. doi: 10.1037/0033-295X.97.3.332.
7. Szũcs D, Soltész F, Jármi É, Csépe V. The speed of magnitude processing and executive functions in controlled and automatic number comparison in children: An electro-encephalography study. Behavioral and Brain Functions. 2007;3:23. doi: 10.1186/1744-9081-3-23.
