# Supplementary material for: A neural network model of mathematics anxiety: The role of attention
Source: PLoS One. 2023 Dec 14;18(12):e0295264. doi: 10.1371/journal.pone.0295264 (PMC10721013; doi:10.1371/journal.pone.0295264)
Supplement: S2 File — (PDF) [file pone.0295264.s002.pdf]

S2 Simulation of the speed-accuracy trade-off during performance of the numerical Stroop task

Procedure

The speed-accuracy trade-off is a well-known and robust effect where decisions made more slowly have increased accuracy than those made in a shorter response time. This simulation demonstrates the speed-accuracy trade-off during performance of the numerical Stroop task. When one of the left or right response nodes in the numerical Stroop model reaches an activation threshold of 0.75 the model records the number of time steps as the simulated response time. To simulate the speed-accuracy trade-off, the value of the activation threshold parameter in the model was adjusted. Arbitrary values of 0.65, 0.70, 0.75, 0.80, and 0.85 were simulated. Reducing the activation threshold parameter has the effect of reducing the simulated response time, and conversely increasing the activation threshold parameter has the effect of increasing the simulated response time. The number of learning trials was 100,000.

Results

For each of the model simulations the percentage of errors was calculated. Figure S2 shows the speed-accuracy trade-off where accuracy increases as the simulated response threshold increases. Some studies have noted speed-accuracy trade-offs while solving calculation problems in individuals with math anxiety, which may have occurred due to avoidance of the numerical stimuli [1].

Fig S2. Model simulations of the speed-accuracy trade-off during the numerical Stroop task

Simulated models' results showing the percentage of errors (on the y-axis) for different values of the response activation threshold (on the x-axis). Error bars depict the standard error of the mean.

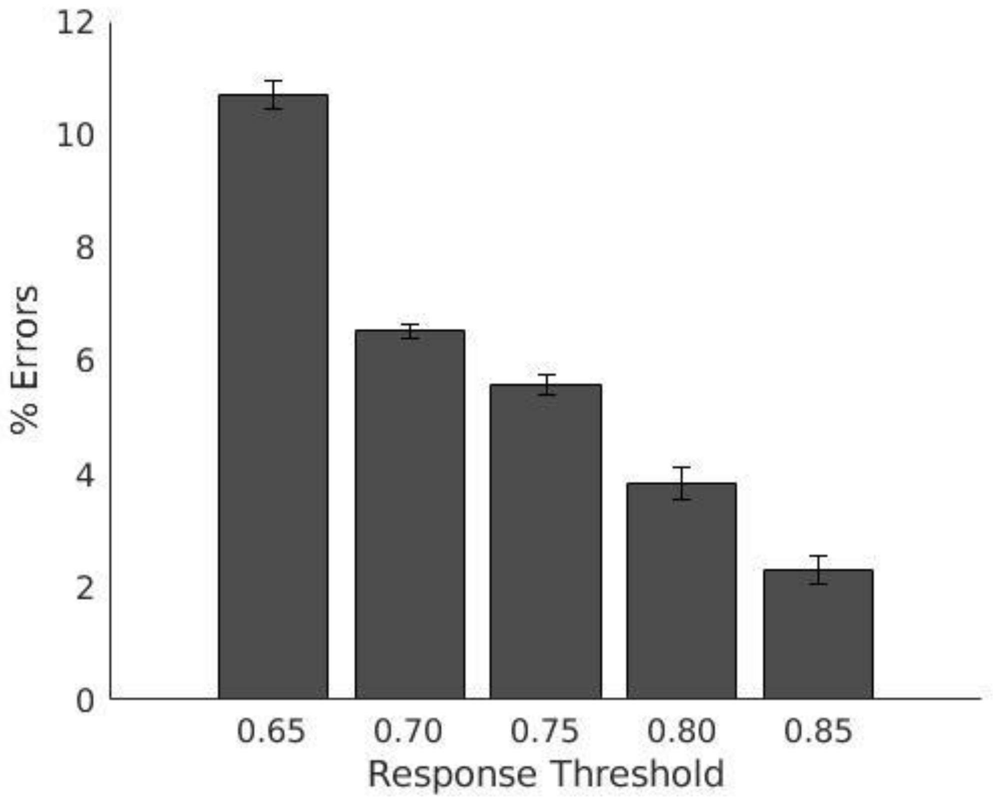

References

1. Faust MW, Ashcraft MH, Fleck DE. Mathematics anxiety effects in simple and complex addition. Mathematical Cognition. 1996;2(1):25–62. doi: 10.1080/135467996387534.
