# Supplementary material for: Correction: A neural network model of mathematics anxiety: The role of attention
Source: PLoS One. 2025 Aug 26;20(8):e0331062. doi: 10.1371/journal.pone.0331062 (PMC12380342; doi:10.1371/journal.pone.0331062)
Supplement: S2 File — (PDF) [file pone.0331062.s002.pdf]

RESEARCH ARTICLE

A neural network model of mathematics anxiety: The role of attention

Angela C. Rose[1], **Hany Alashwal**[2*], **Ahmed A. Moustafa**[3,4], **Gabrielle Weidemann**[1]

1 School of Psychology, Western Sydney University, Sydney, New South Wales, Australia, **2** College of Information Technology, United Arab Emirates University, Al-Ain, United Arab Emirates, **3** Department of Human Anatomy and Physiology, the Faculty of Health Sciences, University of Johannesburg, Johannesburg, South Africa, **4** School of Psychology & Centre for Data Analytics, Faculty of Society and Design, Bond University, Gold Coast, Queensland, Australia

* halashwal@uaeu.ac.ae

Check for updates

OPEN ACCESS

Citation: Rose AC, Alashwal H, Moustafa AA, Weidemann G (2023) A neural network model of mathematics anxiety: The role of attention. PLoS One 18(12): e0295264. https://doi.org/10.1371/journal.pone.0295264

Editor: Tom Verguts, Universiteit Gent, BELGIUM

Received: April 27, 2023

Accepted: November 17, 2023

Published: December 14, 2023

Copyright: © 2023 Rose et al. This is an open access article distributed under the terms of the Creative Commons Attribution License, which permits unrestricted use, distribution, and reproduction in any medium, provided the original author and source are credited.

Data availability statement: The data set and the Matlab source code have been uploaded to the public repository ModelDB with ascension number 2014833. There are no restrictions on the data set. The Matlab code used for the current study was downloaded and adapted from the supplementary materials of Huber S, Nuerk H-C, Willmes K, and Moeller K. A General Model Framework for Multisymbol Number Comparison. Psychological Review. 2016;123(6):667-95. doi:10.1037/rev0000040.

Abstract

Anxiety about performing numerical calculations is becoming an increasingly important issue. Termed *mathematics anxiety*, this condition negatively impacts performance in numerical tasks which can affect education outcomes and future employment. The disruption account proposes poor performance is due to anxiety disrupting limited attentional and inhibitory resources leaving fewer cognitive resources for the current task. This study provides the first neural network model of math anxiety. The model simulates performance in two commonly-used tasks related to math anxiety: the numerical Stroop and symbolic number comparison. Different model modifications were used to simulate high and low math-anxious conditions by modifying attentional processes and learning; these model modifications address different theories of math anxiety. The model simulations suggest that math anxiety is associated with reduced attention to numerical stimuli. These results are consistent with the disruption account and the attentional control theory where anxiety decreases goal-directed attention and increases stimulus-driven attention.

Introduction

The term *mathematics anxiety* has been defined as "a feeling of tension, apprehension, or even dread that interferes with the ordinary manipulation of numbers and the solving of mathematical problems" [1, p.98]. Approximately 31% of fifteen-year-old students across OECD countries reported they become very nervous when doing mathematics problems [2]. Math anxiety negatively impacts performance in numerical and mathematical tasks [3]. The importance of STEM education and mathematics skills to the future of work is increasingly being recognised, and it is likely that students will be entering a very different work force in 2030 [4]. Given the importance of numerical and mathematical skills, understanding and treating math anxiety is essential to improving education and employment outcomes. In this article, we provide the first neural network model of math anxiety, initially focusing on its cognitive correlates, such as attention and inhibition.

Reproduced with permission from American Psychological Association. No further reproduction or distribution is permitted."

Funding: The author(s) received no specific funding for this work.

Competing interests: The authors have declared that no competing interests exist.

Inhibition, attention, and anxiety

The disruption account (also referred to as the debilitating anxiety theory) has been proposed to explain the negative link between math anxiety and mathematics achievement (for reviews see [5, 6]). The theory proposes math anxiety affects processing and recall by impairing attentional and inhibitory functions leaving fewer cognitive resources available for the current task. Learning may also be affected from avoidance of mathematics situations. Attentional control theory (ACT; [7, 8]) can further explain the negative effect of anxiety on task performance, proposing that anxiety increases the influence of the bottom-up stimulus-driven attentional system (responsible for responding to salient or unexpected stimuli; [9]), and decreases the influence of the top-down goal-directed attentional system (responsible for top-down selection of stimuli and responses; [9]), making it difficult to inhibit distracting or irrelevant information to the task at hand. Eysenck et al. [8] suggested the effect of distracting information occurs for both external (such as task irrelevant sensory stimuli), and internal (such as anxious thoughts) stimuli. Furthermore, the authors hypothesized that distracting information primarily impairs processing efficiency (i.e., the effort required to achieve the desired goal, typically measured in response times) rather than performance effectiveness (i.e., the ability to perform the task, typically measured as response accuracy). That is, anxious individuals exert increased effort (for example, they may take longer to perform the task) to counter distraction to attain a comparable quality of task performance (for example, achieve similar error rates) compared to less anxious individuals.

The Stroop task and math anxiety

The Stroop task is a test of cognitive control assessing the ability to inhibit irrelevant information [10]. The Stroop task measures attentional capture, and thus how anxiety may affect attention and top-down inhibition of attention [11, 12]. Studies implementing a colour-word version, in which participants have to inhibit the word, which is a colour label, to name the colour of the ink in which the word is written, have found that interference is greater under conditions of anxiety and stress [13, 14]. Research on individuals with math anxiety suggests that they may have trouble inhibiting attention to distracting information [11]. A math-related Stroop paradigm employed to investigate the effect of math anxiety on inhibitory deficits found that individuals who were high in math anxiety had longer response times in a letter and numerical counting task than individuals who were low in math anxiety, and this difference between the groups was more pronounced in the numerical than the letter condition. However, response times did not differ between the low math-anxious (LMA) and high math-anxious (HMA) groups on a modified Stroop colour-word task for either mathematical or neutral words. This suggests that math anxiety may influence attention and inhibitory control of task irrelevant information, particularly for more salient (i.e., numerical) stimuli.

Suárez-Pellicioni, Núñez-Peña and Colomé [12] used the event-related potentials (ERP) technique to understand whether math anxiety is related to early (i.e., detection) or late stages of the processing of conflict. Participants were tested on a numerical Stroop paradigm, in which two single-digit numbers are presented in different physical sizes and participants must decide which number is numerically larger (see Fig 1). Conflict occurs where the physical size is mismatched with the numerical size and needs to be inhibited. The size congruity effect, or numerical interference effect, is observed where it is easier to decide which number is numerically larger, when this number is also physically larger (the congruent condition), than when this number is physically smaller (the incongruent condition). Suárez-Pellicioni, Núñez-Peña and Colomé [12] found that individuals with math anxiety showed larger numerical interference effects on response times. This result is consistent with an impaired inhibition

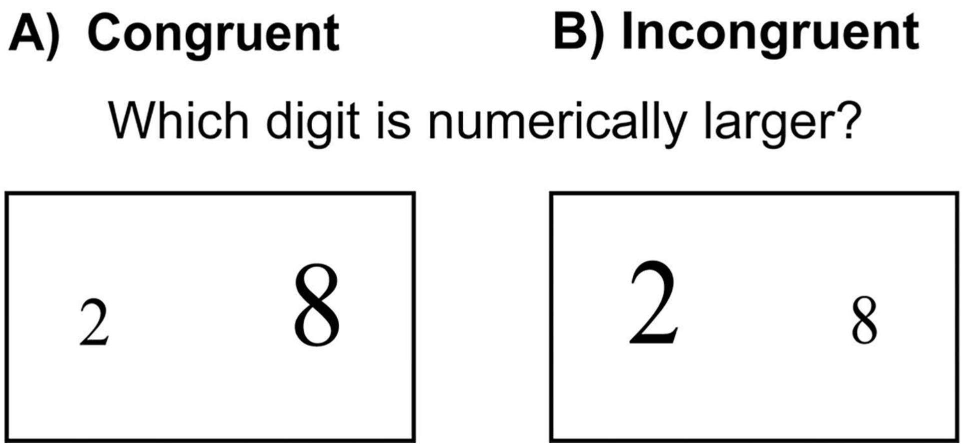

Fig 1. Congruent and incongruent stimuli for the numerical Stroop task. (A) An example of a congruent trial in the numerical Stroop task, when the numerically larger number is also physically larger, and hence there is no conflict between numerical and physical size. (B) An example of an incongruent trial when the numerically larger number is physically smaller and hence there is conflict between the numerical and physical size.

https://doi.org/10.1371/journal.pone.0295264.g001

mechanism and the claims of ACT as it relates to math anxiety. Specifically, it suggests that individuals with math anxiety are more easily distracted by task irrelevant, stimulus driven features of the environment. Additionally, those with the greater level of math anxiety showed the largest numerical interference effects. In contrast, there were no differences between groups in accuracy identifying the numerically larger item, which is consistent with the hypothesis that anxiety impairs how efficient the task is performed (i.e., processing efficiency) to a greater extent than the level of performance (i.e., performance effectiveness).

Other studies have found individuals with math anxiety show deficits in inhibitory abilities [15, 16] and in attentional control [17–20]. In an attentional deployment paradigm assessed using functional magnetic resonance imaging (fMRI), Pizzie and Kraemer [20] observed that math anxiety is associated with attentional disengagement (threat avoidance) specific to numerical stimuli. Ashkenazi [17] investigated the effect of intentional versus automatic processing in individuals with math anxiety by combining a numerical Stroop paradigm with emotional priming with mathematically related words. Participants were tested on separate tasks where the physical size was the irrelevant dimension and when the numerical size was the irrelevant dimension. The author found a larger interference effect among individuals with math anxiety when physical size was irrelevant (as in Suárez-Pellicioni, Núñez-Peña and Colomé [12]) but not when numerical size was irrelevant. This aligns with ACT where anxiety reduces attentional control to the intended dimension and increases attention to the irrelevant (and more automatic) dimension.

Number/magnitude comparison and math anxiety

To investigate the effects of math anxiety on basic numerical skills, performance on magnitude comparison tasks for symbolic quantities have been assessed (e.g., deciding which of two Arabic digits is numerically larger). The number comparison task is related to the underlying hypothesis that numbers are mentally represented as a number line [21]. The numerical distance effect and the size effect are robust findings observed in magnitude comparison tasks. The numerical distance effect is the greater ease in deciding which number is larger when the numbers are further apart than when they are closer together [22]. For example, it is easier to decide which number is larger when comparing 2 and 9 than when comparing 5 and 6. It is

theorised that the numerical distance effect indexes the overlap of numerical representations on the mental number line where proximal magnitudes share greater representational overlap than those that are further apart. This overlap results in numbers that are closer together being harder to discriminate. Another theory regarding the origins of the numerical distance effect in numerical comparisons is that it indexes comparison processes between the numerical stimuli representations and the response [23–25].

The size effect is the greater ease in deciding which number is larger where there is a matched numerical distance, when the numbers are small than when they are large [22]. For example, it is easier to decide which number is larger when comparing 1 and 2 than when comparing 8 and 9. It is theorised that the size effect is due to the overlap between the numerical magnitude representations which increases with numerosity such that larger numbers are represented more vaguely than smaller numbers and are therefore more difficult to discriminate [26]. However, Verguts, Fias and Stevens [25] proposed an alternative explanation for the size effect that it can be explained as (response-related) comparison processes because of the frequency of numbers experienced in daily life where larger numbers occur with a lower frequency.

The numerical distance and size effects in a symbolic number comparison task have been studied in participants with math anxiety with mixed results. Maloney, Ansari and Fugelsang [27] found that differences in response times consistent with the numerical distance effect were more pronounced in the HMA group than for the LMA group. However, there were no overall differences between the groups for response times or error rates. Núñez-Peña and Suárez-Pellicioni [28] investigated the effects of the symbolic number comparison task on individuals with math anxiety, by presenting stimuli as extreme as possible to participants. They found only marginal differences between the groups for both the numerical distance effect and the size effect. Overall, the HMA group were slower than the LMA group but there were no differences between groups in error rates. Dietrich et al. [29] tested participants on a symbolic comparison task in which all combinations of Arabic digits from 1 to 9 were presented. They found a significant association between the numerical distance effect and math anxiety for response times, as in other studies [27, 28]. They also found no significant association between math anxiety and overall response times or the size effect, and no differences in error rates. Colomé [30] examined performance on several numerical comparison tasks. For the symbolic number comparison task, no differences were found between the LMA and HMA groups for overall response times, distance and size effects, or for error rates.

The larger distance and size effects amongst those with math anxiety would suggest they do not have well differentiated numerical representations. However, Dietrich et al. [29] proposed an alternative theory to explain the findings that math anxiety is associated with these larger effects. Individuals with math anxiety may have impaired comparison processes instead of an impaired representation of numerical magnitude, as the numerical distance effect and the size effect can reflect comparison processes between the symbolic representation and the response in deciding which number is larger [23–25]. Impaired comparison processes may be due to less training of the connection between the representation and the "which numeral is larger" response. Individuals with math anxiety may have less trained connections because they may be less motivated to perform or more motivated to avoid numerical calculations. This conclusion fits into the model by Ashcraft, Krause and Hopko [31] who proposed that deficits in basic numerical skills or low motivation may be risk factors in developing math anxiety. Colomé's [30] results further supported this conclusion. However, the author noted that the lack of findings in their study could also be related to the proposal that individuals high and low in math anxiety could differ in attentional control.

Prior neural network modelling

Although there are no neural network models of math anxiety, neural network modelling has been used extensively to investigate the mechanisms of numerical cognition and cognitive control. Below, we discuss these relevant models.

Models of number comparison. In their seminal research article, Verguts, Fias and Stevens [25] implemented a neural network model that proposed a place-coding system to explain how number-selective neurons, that are attuned to numbers, are represented on the mental number line for symbolic numbers. Their model, called a model of exact small-number representation, proposed that the representation of numerical magnitude for symbolic numbers has place coding, linear scaling, and constant variability properties. Verguts, Fias and Stevens' [25] place-coding model simulated several tasks including a symbolic number comparison task. This task involved deciding which of two numbers (from 1 to 15) has greatest magnitude. When two numerical stimuli, presented on the left and right, were input to the model for comparison as to which was largest, the model activated either the left or right response unit corresponding to the left or right input stimuli, depending on which number was the largest. The model has been seminal in developing subsequent neural network models of numerical cognition.

Moeller et al. [32] investigated how two-digit numbers are represented and proposed a separate mental number line that is recycled for each place-value. Their model was extended to resolve conflict which may arise between tens and units in a two-digit number comparison [33]. A conflict-modulated Hebbian learning rule showed the cognitive control system where to intervene when it detected conflict [34]. Subsequently, Huber and colleagues created one general framework for multi-symbol number comparison [35] that integrated their previous models and included comparison of three-digit numbers [36], decimals [37], and negative numbers [35]. The resulting model was validated by simulating most of the standard empirical effects for the number comparison task (e.g., the distance effect).

Models of the distance and the size effects. Neural network modelling has been influential in describing the origins of the numerical distance effect and the size effect. Verguts, Fias and Stevens' [25] neural network model investigated the origins of the distance effect and the size effect for symbolic numbers, which can be explained by different assumptions about how numerical information is represented and processed. The authors proposed a unified framework that accounted for previous empirical findings for the distance effect and the size effect appropriately across different tasks. Furthermore, they proposed an alternative to account for the origins of the size effect in the symbolic number comparison task. Numbers were presented to the model during the training phase with the frequencies that they occurred in daily life, where smaller numbers were presented more often than larger numbers. The model simulations suggested that the size effect for the symbolic number comparison task was the result of comparison processes (i.e., nonlinear mappings) between the mental number line and the output fields. These mappings were derived from the frequency that numbers were presented to the model during learning. Importantly, Verguts, Fias and Stevens [25] explained the origins of the numerical distance effect and the size effect in the symbolic number comparison task as developing from the monotonicity (the condition of consistently increasing or decreasing in value) of the connection weights between the stimuli and the response units. van Opstal et al. [23] and van Opstal and Verguts [24] further examined distance effects by neural network modelling proposing that the distance effect has different origins depending on the task context. Specifically, they showed that the "comparison" distance effect that is obtained from a symbolic number comparison task is derived from comparison processes between the stimuli and response and not from the overlap of the numerical representations of the stimuli.

Models of the Stroop task and conflict monitoring. Santens and Verguts' [38] neural network model of the numerical Stroop task implemented a dual route architecture to simulate the shared decisions account, proposing that numerical size and physical size are initially processed separately then interact at the decision level of the task. The colour-word version of the Stroop task has been modelled previously by Botvinick et al. [39] who implemented a series of neural network models to propose how the cognitive control system detects the need to intervene when it encounters conflict. Botvinick et al.'s [39] conflict monitoring hypothesis proposed that a conflict monitoring system evaluates the amount of conflict in the system and consequently regulates the amount of top-down cognitive control based on current task demands. The authors hypothesised that the detection of conflict may be a function of the anterior cingulate cortex (ACC) based on data from empirical studies. In a series of simulations, they extended existing neural network models to include a conflict monitoring unit that calculated the amount of response conflict at each step of processing. Conflict in the models was defined as the simultaneous activation of incompatible (i.e., alternative) responses of mutually inhibiting units. In subsequent simulations, Botvinick et al. [39] created a feedback loop from the conflict monitoring unit, to use the amount of conflict detected on previous trials as a signal to adjust top-down control. When a large amount of conflict was detected, cognitive control was strengthened. Conversely, if a low amount of conflict was detected, cognitive control was weakened. Furthermore, recent research has seen the conflict monitoring hypothesis under debate. Other cognitive mechanisms, such as learning and memory biases, have been proposed to explain the various congruency effects either alternatively or in conjunction with conflict monitoring and adaptation (see Schmidt [40] for a review).

The current study

It is well established that math anxiety negatively impacts performance in numerical tasks. However, the cognitive mechanisms underpinning this are not understood. The aim of the current research is to investigate whether poor performance due to math anxiety is due to impaired attentional and inhibitory functions (as suggested by the disruption account) by using neural network modelling. ACT's claims of how anxiety influences task performance will be used as a framework. Math anxiety has primarily been studied in behavioural experiments, and more recently using brain imaging and electrophysiological recording techniques. However, to the best of our knowledge, there have been no studies simulating math anxiety with neural network modelling. We have previously specified a novel and theoretical neural network model of numerical processing with modifiable inhibition and attentional processes to simulate the neural and behavioural studies of math anxiety [41]. The current study describes an initial implementation of specific aspects of this model. Our neural network model will investigate the effect of specific impairments on the outcomes of two different experimental tasks: numerical Stroop and symbolic number comparison. The model is novel as it integrates Santens and Verguts' [38] numerical Stroop neural network model into Huber et al.'s [35] multi-symbol number comparison framework by adapting Huber et al.'s [35] cognitive control network that simulates Stroop-like effects. The modified architecture allows investigation of the different cognitive mechanisms and possible impairments that may underlie math anxiety, not only the processes examined in the current research. The adapted model is validated to ensure it simulates various standard aspects of numerical processing including the standard findings from both tasks thus establishing a reasonable model of an individual without math anxiety. The effects of specific impairments are simulated and compared to research from participants with math anxiety.

We have investigated whether math anxiety impairs the inhibition function during numerical tasks by modelling the numerical Stroop task as it requires inhibition of task irrelevant

stimuli. Previous studies of this task have suggested inhibitory deficits in math-anxious individuals [11, 12]. Inhibitory parameters are not modified in our model, instead we have focused on investigating impairments of attention by modifying the parameters in the model associated with attentional control. Our adapted model specifies top-down goal-directed attention and bottom-up stimulus-driven attention via setting task demands associated with the relevant and irrelevant dimension of the task respectively. ACT suggests that anxious individuals have an imbalance between the top-down goal-directed and the bottom-up stimulus-driven attentional systems where anxiety is associated with reduced top-down attention. Reduced top-down attention will impair the ability to inhibit distracting or irrelevant information. To model this, we modulated the amount of attention in these two attentional systems and investigate the effect on simulated task performance. Furthermore, ACT proposes that anxiety affects processing efficiency (where effort to perform the task is measured by response times) to a greater extent than performance effectiveness (where effectiveness is measured by accuracy). We investigate the effect of impaired attention in our model on these measures. A high math-anxious model with attention impaired will be compared to a low math-anxious model without attention impaired to investigate differences in the amount of energy in the response layer (i.e., conflict) during congruent and incongruent trials.

We simulated the effect of reduced attention on the single-digit symbolic number comparison task to investigate its effect on basic numerical skills. Results of studies are inconsistent, and Colome [30] noted that the lack of differences between individuals with and without math anxiety in their study could be due to differences in attentional control. Furthermore, Dietrich et al. [29] suggested that individuals with math anxiety may have less trained connections between the numerical representation and the response (i.e., impaired comparison processes) to explain the larger distance effects associated with math anxiety in some studies. Recent research has shown that the distance effect obtained in symbolic number comparison reflects comparison processes between the symbolic representation and the response and does not reflect the numerical representation. Therefore, we investigated the effect of reduced training by reducing the parameter specifying the amount of learning that the model performs. This parameter reflects how much the model trains the connection weights between the numerical stimuli and the response when comparing two numbers and deciding which number is the largest. Verguts, Fias and Stevens [25] explained the origins of the distance and size effects as developing from the monotonicity of these connection weights.

Method

This section describes the neural network architecture used for modelling numerical representations and cognitive processes used in the current research. More details about the model, including equations, are in S1 File.

Model architecture

The integrated framework for the comparison of multi-symbol numbers developed by Huber et al. [35] was adapted to simulate the numerical Stroop task (see Fig 2 for schematic illustration). Huber et al.'s [35] model simulates Stroop-like effects of two-digit number comparison where the comparison of the tens digits have more relevance than the comparison of the units digits. Consequently, numerical size and physical size from the numerical Stroop model of Santens and Verguts [38] were each mapped onto the tens and units modules of the Huber et al. [35] model, respectively. This mapping facilitates the Stroop effect in the numerical Stroop task where numerical size has more relevance than physical size. The model uses a dual route architecture to reflect the shared decisions account (as in the study of Santens and Verguts

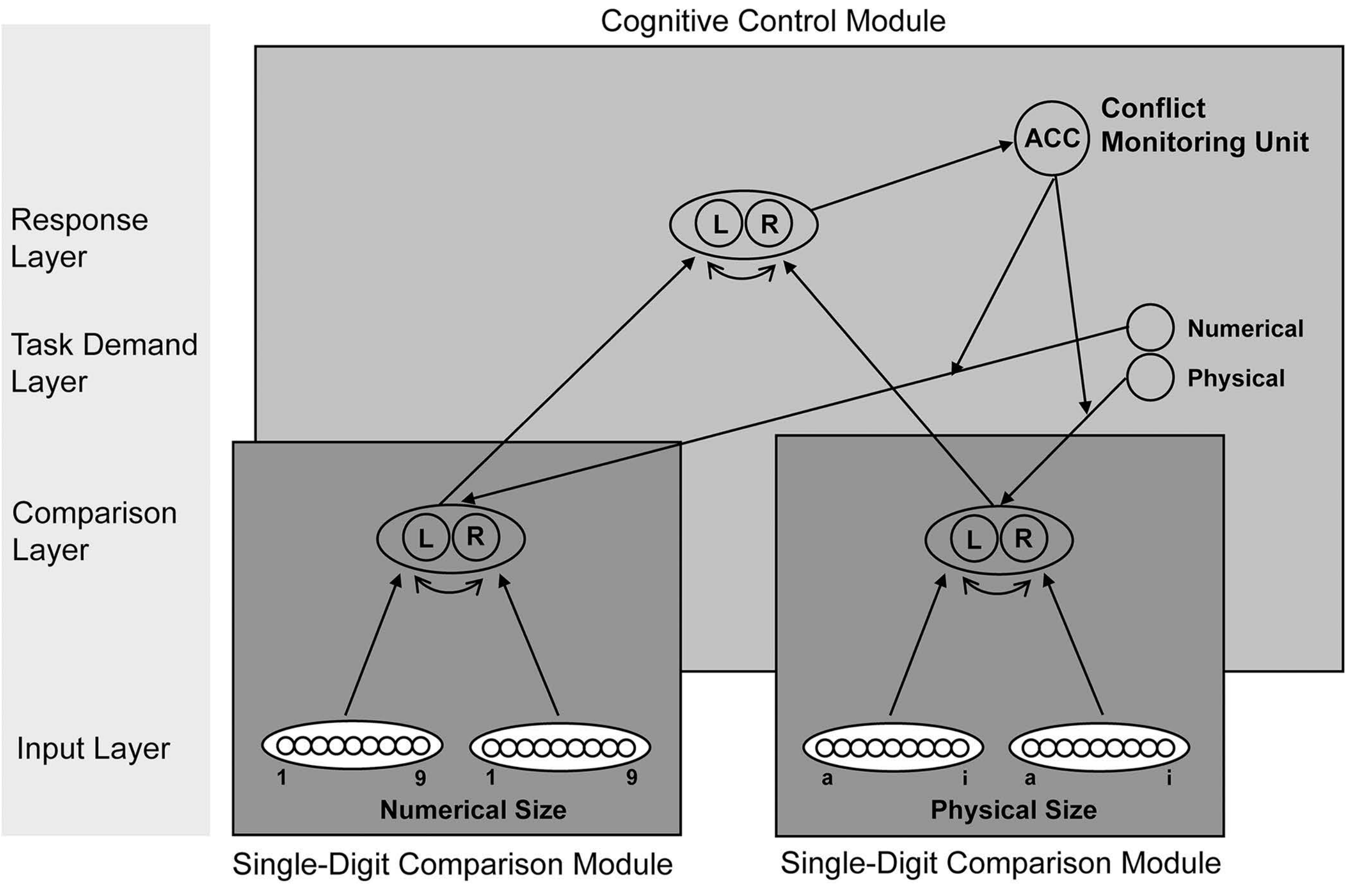

Fig 2. Schematic illustration of the neural network model architecture. Schematic illustration of the neural network model architecture for the simulation of the numerical Stroop task and the symbolic number comparison task. The model consists of two single-digit comparison modules, one for the numerical size and one for the physical size. The task demand units serve as an attentional bias to specify the relevant and irrelevant dimension of the task. Information is propagated to the response layer where the model decides whether the left (L) or right (R) input number is the largest. The conflict monitoring unit calculates the amount of conflict during the task and adjusts attention accordingly. ACC refers to the anterior cingulate cortex.

https://doi.org/10.1371/journal.pone.0295264.g002

[38]) where numerical size and physical size only interact at the decision level. The model consists of four layers: an input layer, comparison layer, task demand layer, and a response layer; and includes a cognitive control module as implemented by Verguts and Notebaert [34] and adapted by Huber et al. [33] and Huber et al. [35]. Additionally, features of the numerical Stroop model for magnitude judgment implemented in Experiment 1 of the study by Santens and Verguts [38] were incorporated into the adapted architecture. The programming code written in Matlab by Huber and colleagues was downloaded from the supplementary materials of Huber et al. [35]. The code is reproduced with permission from American Psychological Association. No further reproduction or distribution is permitted.

Single-digit comparison module

The input layer consists of a single-digit comparison module each for numerical size and physical size. The numerical size module contains two number line fields that code the representation of numerical magnitude for the left and right Arabic digits to be compared that are presented to the model. Each number line field is implemented as in Santens and Verguts [38] and is a vector of nine nodes. Each of the nine nodes represent one Arabic digit to create an ordered sequence of natural numbers, allowing for the comparison of sizes 1 to 9. A number

line field represents numerical magnitude using a place-coding system with linear scaling and constant variability as in the model of exact small number representation by Verguts, Fias and Stevens [25] (see also [35, 38]). The single-digit number comparison module for physical size is represented identically to that of numerical size. Nine physical sizes (a—i) are mapped onto each of the nine number line nodes respectively as in the model of Santens and Verguts [38]. The equation for the activation of the number line nodes is in S1 File.

Propagation of input to comparison layer

The propagation of the input layer to the comparison layer is identical to the study of Huber et al. [35]. All nodes in the number line fields for a single-digit comparison module are propagated via feed forward connections to all nodes in the hidden comparison layer for that module. The comparison layer for each single-digit comparison module consists of a left and right node coding for "left larger" or "right larger" (see S1 File for details).

Training of weights between input and comparison layers

Prior to running the simulations, the weights between the input and comparison layers of the single-digit comparison modules were trained (see S1 File for further details). Training occurred identically to Huber et al. [35] with the exception that the numerical and physical size comparison modules were trained independently (as in Santens and Verguts [38]) as they are independent of each other in the numerical Stroop task. Each single-digit number comparison module was trained to compare all combinations of single-digit numbers from 1 to 9 with the exception of the numbers being equal. The frequency of each number presented to the model during training was taken from a Google survey which observes the frequency of numbers observed in daily life and allows simulation of the problem size effect (see also [25, 42]). The model was trained for 100,000 trials (chosen arbitrarily) to ensure all combinations were compared correctly (as in Huber et al. [35]).

Cognitive control module

The output of the single-digit comparison modules serves as input to the cognitive control module (further details are in S1 File).

Task demand layer. The numerical size and physical size single-digit comparison modules have feed forward connections to the response layer. Activation from the comparison layer to the response layer is modulated by the task demand layer which comprises of two nodes, one node for numerical size and one node for physical size. The task demand layer serves as an attentional bias [39, 43] to specify the relevance of the numerical size dimension and irrelevance of the physical size dimension in the numerical Stroop task. The stronger the activation of the task demand nodes, the more relevant the dimension and greater the influence on the comparison process. The task demand nodes are also associated with top-down goal-directed attention and bottom-up stimulus-driven attention for the relevant (i.e., numerical size) and irrelevant (i.e., physical size) dimensions respectively. The activation of the numerical size task demand node is set at 1.0 and the activation of the physical size task demand node is set at 0.15 allowing for more attention directed to the relevant numerical size dimension. These values are identical to the amount of attentional bias of the relevant and irrelevant dimensions respectively of the numerical Stroop model of Santens and Verguts [38].

Response layer. The response layer is implemented as in Huber et al. [35]. A left and a right node code for the response "left larger" or "right larger" respectively. The nodes have lateral inhibitory connections between them with w^{inh} = -0.5 that cause response competition and reduce the amount of time taken for the model to make a decision. When the activation

of one of the response nodes reaches the prespecified threshold parameter theta, the model records the number of time steps t to reach that decision as the simulated response time. If the left node reaches the threshold value first, then the model has decided that the left input stimulus number has the largest numerical size while ignoring its physical size. If the right node reaches threshold first, then the right input stimulus number has the largest numerical size while ignoring its physical size. The value of the threshold parameter θ is 0.75. A maximum number of time steps t is set at 200 in case the activation threshold is not reached. Similar to the comparison layer, the response layer calculates a weighted sum of activation over time with a constant value of $\tau = 0.25$ as the rate of activation that impacts the amount of time it takes to reach the response unit threshold.

Conflict monitoring unit. As in the models of Huber et al. [33] and Verguts and Notebaert [34] a conflict monitoring unit calculates the amount of conflict during a trial as the energy in the response layer which is calculated by the product of the activation of the response nodes [39]. At the end of each trial, if the level of conflict on the current trial is high compared to previous trials, the conflict monitoring unit can adapt the weights between the task demand layer and the comparison layer via the conflict-modulated Hebbian learning rule as described in equations (A3) and (A4) of Verguts and Notebaert [34]. The learning rule has the effect of strengthening attention to the relevant numerical size dimension and weakening attention to the irrelevant physical size dimension as needed. The current research does not study conflict adaptation effects (i.e., the effect of the previous trial's congruency on the current trial), therefore the conflict monitoring unit does not adapt the weights in the simulations. However, subsequent research could investigate conflict adaptation effects as they relate to math anxiety. The initial weights between task demand nodes and comparison layer nodes were set as 0.5 [33]. All other parameters for the conflict monitoring unit equations remained the same as in Huber et al. [33].

Results and discussion: The numerical Stroop task

Validation of the numerical Stroop model

Before impairing the LMA model to create the HMA model, the LMA model was validated to ensure it can simulate various experimental effects. Below is a description of the simulations of the numerical Stroop task assessing the behaviour of the model on empirical effects for this task. In addition to simulating standard effects of numerical Stoop, reduced learning, the speed-accuracy trade-off (see S2 File for simulation details), and the physical Stroop task were simulated to demonstrate the model's behaviour in other cognitive conditions.

Simulation of the numerical Stroop task. The numerical Stroop neural network model created by Santens and Verguts [38] simulated experimental effects which are reproduced using simulations of the current LMA model. These include the size congruity effect (or interference effect) where it is faster to compare stimulus pairs that are congruent than when they are incongruent, and the numerical distance effect where it is faster to compare stimulus pairs when the distance between the numbers is further apart than when the distance between the numbers is closer together. In these simulations the relevant dimension was the numerical size and the irrelevant dimension was the physical size. The current data set was constructed in a similar way to Experiment 1 of Santens and Verguts [38] where all combinations of four sizes of Arabic digits for both the numerical size and physical size create 12 x 12 = 144 stimulus pairs, excluding instances where the numerical size or the physical size are equal. Congruent stimuli and incongruent stimuli were presented to the model in equal proportions. The four numerical sizes presented to the input layer of the current model were the Arabic digits 1, 2, 8, and 9 as used in the math anxiety study of Suárez-Pellicioni, Núñez-Peña and Colomé [12] for

which the current study's HMA models' results are compared. There were four physical sizes presented to the model a, b, h, and i. These were mapped onto the Arabic digits 1, 2, 8, and 9 for the purposes of the simulation (see Santens and Verguts [38] for a similar approach which used Arabic digits 1, 2, 7, and 8 mapped onto physical sizes a, b, g, and h). Suárez-Pellicioni, Núñez-Peña and Colomé [12] used a reduced set of numerical sizes and physical sizes in their math anxiety study and the data set for the current study was reduced to their data set for the HMA model simulations once the LMA model was validated. The trial-to-trial adaptation of the conflict monitoring unit was turned off. The activation of neurons was reset at the beginning of each trial. Therefore, all 144 trials presented to the model were independent. The model simulated 30 participants who were low math-anxious. Distance effects between the numerical sizes and between the physical sizes were modelled. The distance was classified as small (also termed *close*) when the distance between the numerical sizes or between the physical sizes was 1 or 6. The distance was classified as large (also termed *far*) when the distance between the numerical sizes or between the physical sizes was 7 or 8. This allowed an equal amount of observations at each level (see Santens and Verguts [38] for a similar approach who classified small when the distance between numerical or physical sizes was 1 or 5, and large when the distance between them was 6 or 7).

The results for the LMA model were based on replicating the results from Experiment 1 of Santens and Verguts [38]. The response time for each trial is the number of time steps the model takes to reach a decision. The mean response times for successful trials was calculated in each condition. The size congruity effect was calculated as the mean response time for incongruent trials minus congruent trials. The results of the LMA model simulations are described below and were compared to the response patterns of Santens and Verguts [38] as shown in Fig 3. The main results are as follows: (a) The model was able to simulate the size congruity effect for response times where congruent trials were faster than incongruent trials (see Fig 3B and 3D); (b) The model was able to simulate the numerical distance effect for response times with faster decision times when the numbers were far apart than when they were close (see Fig 3B); (c) Importantly, the model simulated an interaction between the congruity effect and the numerical distance. The congruity effect was larger for a small numerical distance than for a large numerical distance (see Fig 3B); (d) The current LMA model did not produce a difference in mean response times between the physical distance being small or large (see Fig 3D). However, response times were faster when the distance between the physical sizes was small than when it was large in the behavioural data from Santens and Verguts [38] (see Fig 3C); (e) The model simulated an interaction between the congruity effect and the physical distance. The congruity effect was larger for a large physical distance than for a small physical distance (see Fig 3D); and (f) Participants did not respond in time or made an error on 1.8% of trials in Santens and Verguts' [38] behavioural study. In their simulations the model produced 1.5% errors. In both instances the errors were all made in the slower conditions (i.e., incongruent, small numerical distance, large physical distance). The current LMA model produced 5.3% errors that all occurred in the slower conditions where trials were incongruent and the distance between the numerical sizes was small.

Simulation of changes in the amount of learning. This simulation demonstrates the effect of training the connections less between the numerical representation and the response on the numerical Stroop task. The numerical size and physical size single-digit comparison modules were trained with different values for the number of learning trials (see section Model Architecture for a description of the training). This created a different set of weights for each training run. Training was performed such that the connection weights were identical for equal values of the number of learning trials. For example, on each trial, numerical stimuli were presented to the model such that when the training of the model reached 10,000 learning

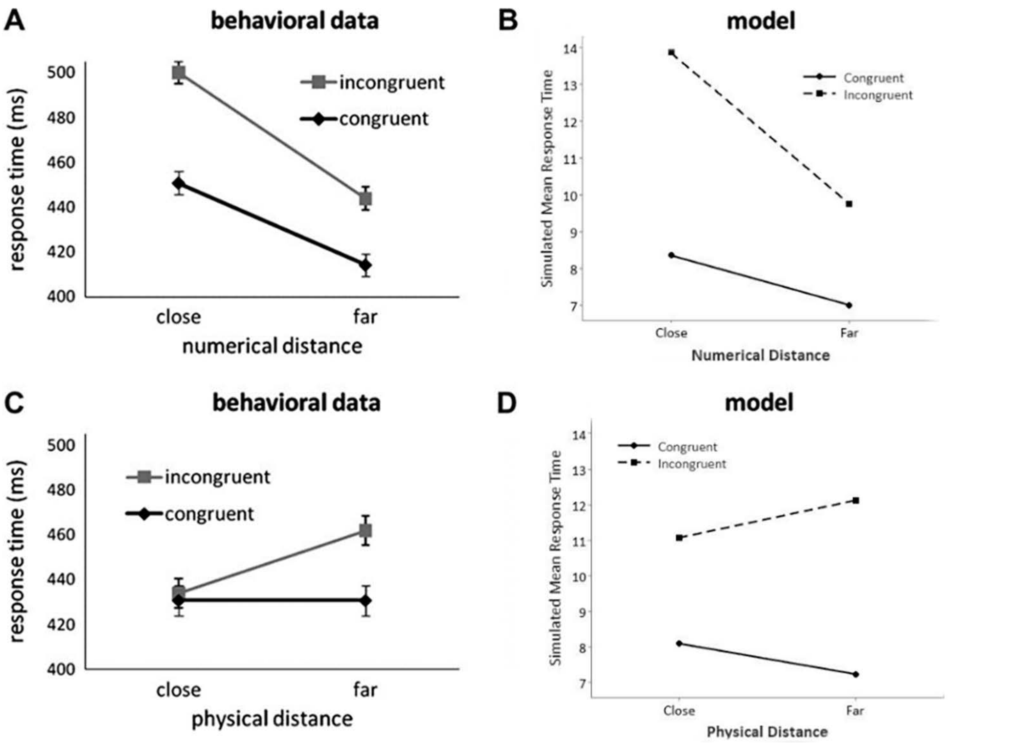

Fig 3. Validation of the numerical Stroop model. Results for the numerical Stroop task with numerical size as the relevant dimension and physical size as the irrelevant dimension. Panels A and C are mean response times of behavioural data from Santens and Verguts' [38]. Panels B and D are the current study's simulated LMA model mean response times. Panels A and B depict the numerical distance and panels C and D depict the physical distance. Error bars for behavioural data represent 95% confidence intervals. Panels A and C: From "The Size Congruity Effect: Is Bigger Always More?" by Santens S, Verguts T. Cognition. 2011;118(1). p. 98. doi: 10.1016/j.cognition.2010.10.014 [38]. Copyright 2010 by Elsevier B.V. Reprinted with permission from Elsevier.

https://doi.org/10.1371/journal.pone.0295264.g003

trials, during the training of a total of 17,000 trials, the connection weights were identical to the end weights of the previous training when the model was trained for a total of 10,000 learning trials. After training, model simulations were then run for these different amounts of learning trials. Larger numbers of learning trials represent increased learning. The mean response times for successful trials was calculated in each condition.

In previous simulations the number of learning trials was 100,000 as in the general model framework of multi-symbol number comparison of Huber et al. [35] which resulted in 100% accuracy for the single-digit comparison modules. In the current simulations, the model predicts that as the amount of learning increases, response times will decrease and errors will decrease (see Fig 4). This result is consistent with empirical research showing that learning of basic numerical processing skills across a variety of different tasks improves response times and accuracy (e.g., Landerl [44]).

Simulation of the physical Stroop task. The physical Stroop task involves deciding which number has the largest physical size while ignoring the numerical value. The relevant and irrelevant dimensions are reversed from the numerical Stroop task. The conditions for the simulations of the physical Stroop task were the same as used to validate the LMA model of the numerical Stroop task but the relevant dimension in the model was physical size and the irrelevant dimension was numerical size. Several simulations were performed to demonstrate the change in the size congruity effect (i.e., the difference between mean response times for incongruent minus congruent trials) for different amounts of training (which were chosen

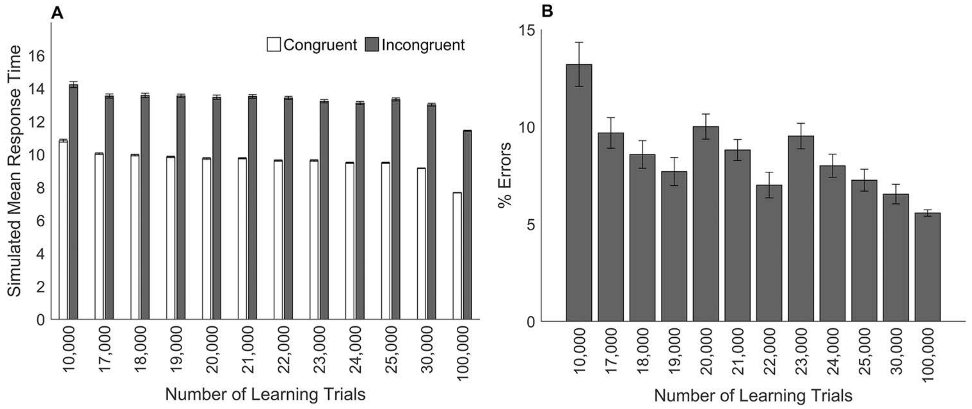

Fig 4. The effect of changing the amount of learning in the numerical Stroop task. Simulated models' results for different numbers of learning trials on performance in the numerical Stroop task. Panel A shows mean simulated response times. Panel B shows the percentage of errors. Error bars depict the standard error of the mean.

https://doi.org/10.1371/journal.pone.0295264.g004

arbitrarily). The size congruity effect was calculated for response times of successful trials in each of the simulations for the different amounts of learning. The congruity by numerical distance was examined for one of the simulations where the number of learning trials was 17,000 (chosen arbitrarily). For this simulation, the simulated mean response time was calculated in the congruent and incongruent conditions by the irrelevant numerical distance.

The results for the simulation of the size congruency effect on response times in the physical Stroop task are shown in Fig 5A. The results of the model suggest that the size congruity effect increases as the training trials increase. These results are consistent with empirical results where the size congruity effect increases as children learn the meaning

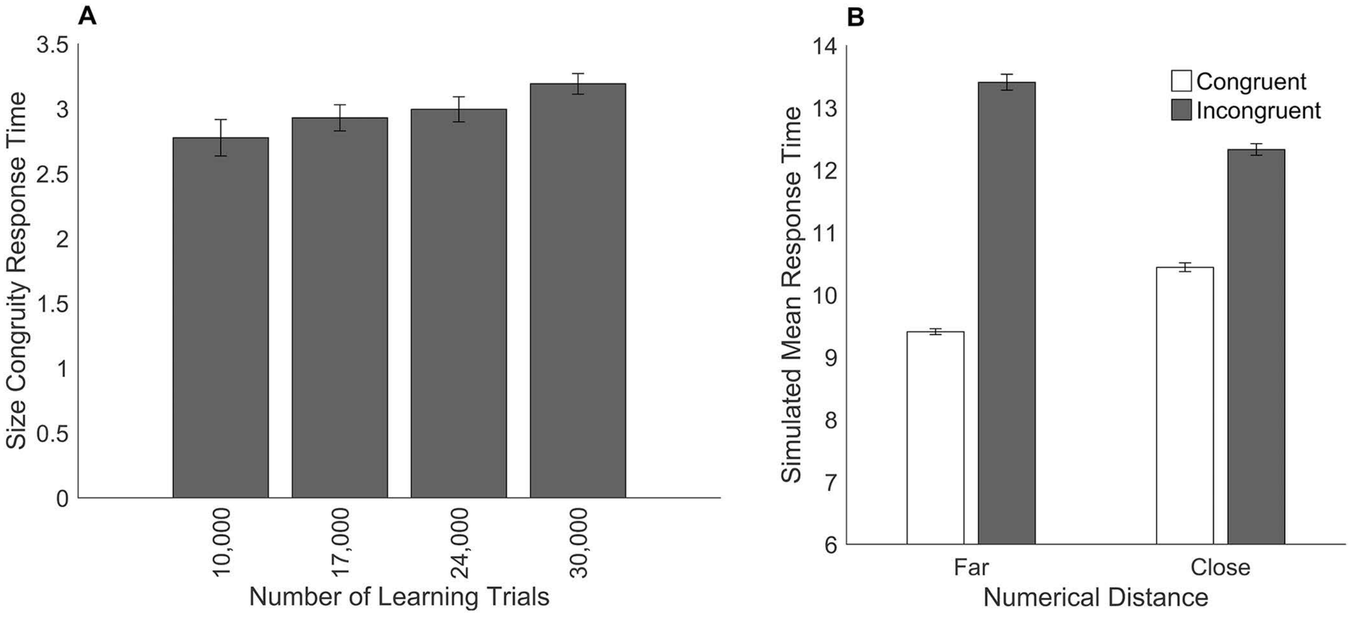

Fig 5. Model simulations of the physical Stroop task. Simulated models' results of the physical Stroop task. Physical size is the relevant dimension. Numerical size is the irrelevant dimension. Panel A shows the size congruity effect for different numbers of learning trials. The y-axis depicts the size congruity effect which is the difference in the simulated mean response times for incongruent trials minus congruent trials. Panel B shows the mean simulated response time for the congruent and incongruent conditions when the distance between the numerical sizes is far and close. Error bars depict the standard error of the mean.

https://doi.org/10.1371/journal.pone.0295264.g005

of symbols (e.g., Landerl and Kölle [45]). The results for the simulation as a function of the differences in numerical size are shown in Fig 5B. In the congruent condition it is faster to decide which number has the largest physical size when the distance between the numerical sizes is large than when the distance between them is small. In the incongruent condition it is faster to decide which number has the largest physical size when the distance between the numerical sizes is small than when the distance between them is large. Furthermore, the size congruity effect is larger when the distance between the numerical sizes is large than when the distance between them is small. These results are consistent with empirical studies examining the speed of processing in the physical Stroop task (e.g., Landerl and Kölle [45]).

Math anxiety experimental results for the numerical Stroop task

The results from the model simulations for the current study aim to reproduce the pattern of results from the math anxiety study by Suárez-Pellicioni, Núñez-Peña and Colomé [12]. These authors tested LMA and HMA participants performing the numerical Stroop task. The data set was reduced to the stimulus pairs used by Suárez-Pellicioni, Núñez-Peña and Colomé [12]. All combinations of the Arabic digit pairs 1–2, 1–8, 2–9, and 8–9 as numerical sizes with two physical sizes (small and large) were presented to the model producing 16 unique stimulus combinations. Physical sizes consisted of small font size 40 presented to the model as number 2, and large font size 80 presented to the model as number 8. The physical sizes presented to the model were chosen arbitrarily. The number of congruent and incongruent trials were presented in equal proportions. All other conditions were the same as in the simulations of the numerical Stroop validation. The single-digit comparison modules for numerical size and physical size were trained separately for 30 simulated participants, so that each simulated participant had a different set of weights. Those weights were subsequently used for each participant for both the LMA and HMA models. For example, the weights for simulated LMA participant 1 were the same as for simulated HMA participant 1. The weights for simulated LMA participant 2 were the same for simulated HMA participant 2, and so on. Keeping the connection weights the same for the LMA and HMA models facilitated a comparison of results.

The mean of response times for successful trials and percentage of hits were calculated for the congruent and incongruent conditions for the LMA and the HMA models. Similar to the study of Suárez-Pellicioni, Núñez-Peña and Colomé [12], a single score index of interference was then calculated from these means by taking incongruent response times minus congruent response times (i.e., the size congruity effect) and congruent percentage of hits minus incongruent percentage of hits (i.e., accuracy). For both measures the larger the score the larger the degree of interference. Suárez-Pellicioni, Núñez-Peña and Colomé [12] did not report results for congruent and incongruent trials separately. However, results were acquired by contacting them. Their results are compared with the current study's model simulation results to determine whether the simulation captures the important differences between LMA and HMA groups. A summary of the central characteristics of the behavioural data are as follows: (a) There were no significant differences in response times between the LMA and HMA groups for congruent trials; (b) The HMA group had significantly longer response times than the LMA group for incongruent trials; (c) The interference effect for responses times was significantly larger for the HMA group than the LMA group; and (d) There were no significant differences in the interference score for accuracy between the LMA and HMA groups. Furthermore, the percentage of hits for the LMA group was 21.62%, and for the HMA group was 20%.

Simulation of the HMA model with impaired attention

Procedure. These simulations test the effect of reduced attentional control during performance of the numerical Stroop task. Model simulations of reduced attention to the numerical size dimension, reduced attention to the physical size dimension, and reduced attention to both the numerical and physical size dimensions were performed. To simulate reduced attentional control, the attention module of the model was impaired by reducing the activation of the neurons in the task demand units for the numerical size or physical size as appropriate.

Results. Fig 6 shows the experimental and simulated findings on the numerical Stroop task. Results from the model simulations of reduced attentional control were compared to the experimental findings of Suárez-Pellicioni, Núñez-Peña and Colomé [12].

Reduced attention to numerical size. The HMA model was simulated with attention reduced to the numerical size dimension and not reduced to the physical size dimension. The results for reducing attention to 95% on the numerical size dimension for the HMA model are reported as this level of impairment was generally consistent with the experimental findings. Percentages close to 95% were also consistent with the above-mentioned experimental findings. The more attention was reduced, the longer the response time. The HMA model produced longer response times than the LMA model in the incongruent condition, which had the effect of increasing the interference effect for response times compared to the LMA model (Fig 6B). The HMA model did not differ from the LMA model on response times for congruent trials or on the size of the interference effect for accuracy. The interference effect for the percentage of hits was similar to the experimental data with the LMA model having a value of 22.08% (experimental was 21.62%) and the HMA model was 20.83% (experimental was 20%). Further analysis of the comparison of the LMA model and the HMA model for response times found that all congruent trials (with the exception of one trial where the distance between the numerical sizes was small) were equal. Furthermore, all incongruent trials that differed between the LMA model and the HMA model were by a simulated response time of 1. The model with reduced attentional control to the numerical size dimension produced results qualitatively consistent with the above-mentioned experimental findings on math anxiety.

Reduced attention to physical size. The HMA model was simulated with attention reduced to the physical size dimension and not reduced to the numerical size dimension. The results for reducing attention to 95% on the physical size dimension for the HMA model have been reported. Results were similar for other values of reduced attention. For response times, the HMA model did not produce a difference to the LMA model in the congruent or incongruent conditions, or for the interference effect (Fig 6C). The model with reduced attention to the physical size dimension did not produce results qualitatively consistent with the above-mentioned experimental findings on math anxiety.

Reduced attention to numerical size and physical size. The HMA model was simulated with attention reduced to both the numerical and physical size dimensions. The results for reducing attention to 95% on both numerical and physical size dimensions for the HMA model are reported. Percentages close to 95% were also consistent with the above-mentioned experimental findings. The more attention was reduced, the larger the response time. The HMA model produced longer response times than the LMA model in the incongruent condition and for the interference effect (Fig 6D). The HMA model did not differ from the LMA model on response times for congruent trials or on the size of the interference effect for accuracy. The model with reduced attention to the numerical and physical size dimensions produced results qualitatively consistent with the above-mentioned experimental findings on math anxiety.

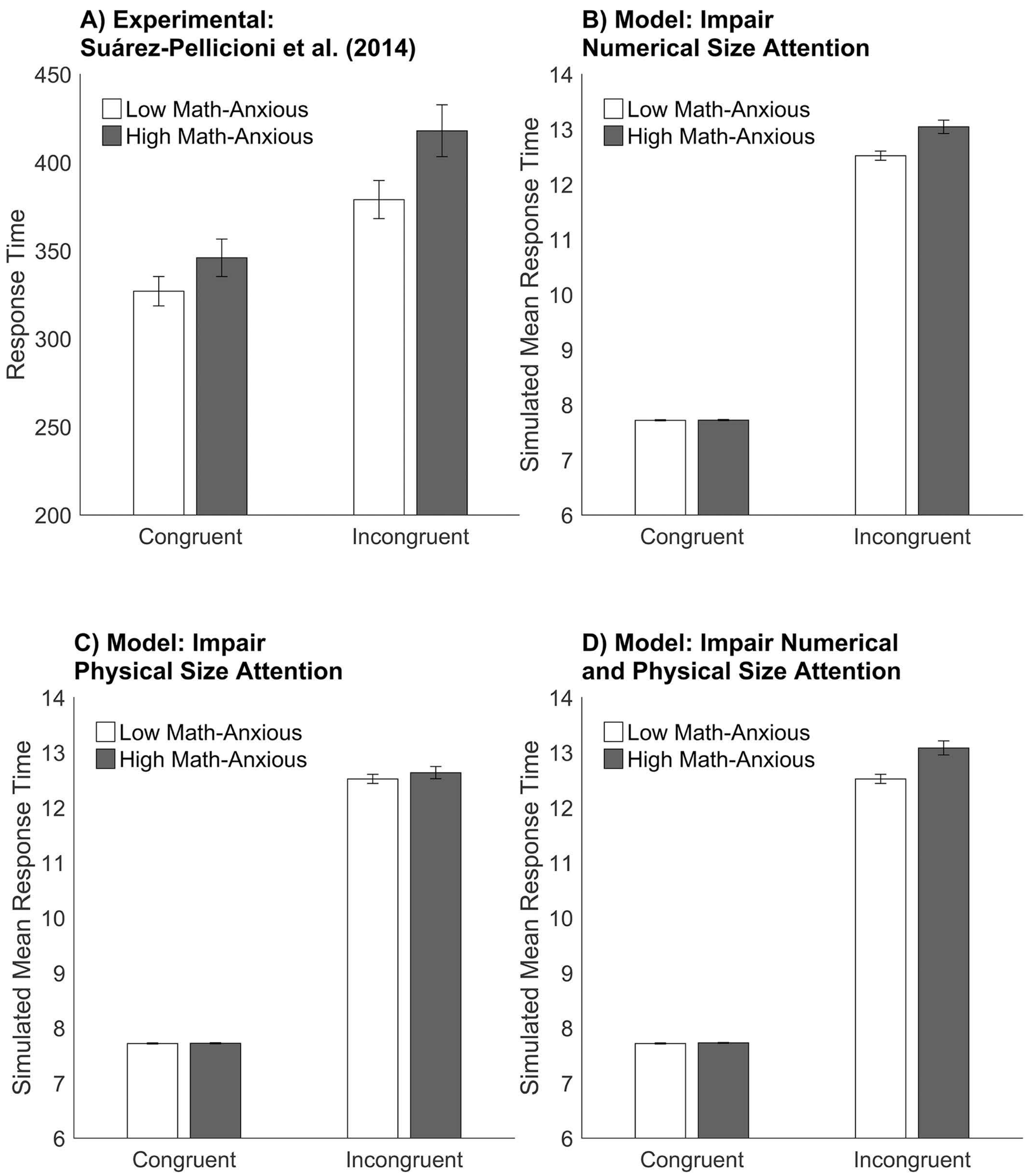

Fig 6. Model simulations of the numerical Stroop task with attention impaired. Mean response times for the numerical Stroop task in the congruent and incongruent conditions for high and low math anxiety are shown for experimental and modelling results. Panel A: Experimental results from Suárez-Pellicioni, Núñez-Peña and Colomé [12]. CC BY 4.0. The y-axis shows the mean of the median of response times. Panels B, C and D: show the results of the model simulations where the high math-anxious model has attention impaired. Panel B: Attention is impaired to the numerical size. Panel C: Attention is impaired to the physical size. Panel D: Attention is impaired to both the numerical and physical sizes. The y-axis shows the simulated mean response time. Error bars depict the standard error of the mean.

https://doi.org/10.1371/journal.pone.0295264.g006

Discussion. The model specifies top-down goal directed attention and bottom-up stimulus-driven attention via setting task demands associated with the relevant (i.e., numerical size) and irrelevant (i.e., physical size) dimension of the task respectively. Results of the simulations suggested that when attention was impaired (i.e., reduced) to the numerical stimuli (either by impairing attention to the numerical size dimension only, or by impairing attention to both the numerical size and physical size dimensions together), that the models' results qualitatively match experimental results on math anxiety. However, when attention was impaired to the non-numerical and irrelevant dimension (by impairing attention to the physical size dimension in the model only), the results did not match those of experimental studies on math anxiety. These results are consistent with previous studies that suggest that math anxiety is associated with reduced attentional control while processing numerical stimuli [17–20]. The HMA model consistent with these findings (with attention reduced to the numerical size dimension only) simulated longer response times in the incongruent condition and for the interference effect than simulated by the LMA model. However, there were no differences in the error rates between these LMA and HMA models. These results are consistent with previous studies where individuals high in math anxiety experience more interference than individuals low in math anxiety [11, 12]. Furthermore, they are consistent with ACT [7, 8] suggesting anxiety reduces the attentional resources of working memory. This results in an increased influence of the bottom-up stimulus-driven attentional system and a decreased influence of the top-down goal-directed attentional system. Consequently, math anxiety is associated with a reduced ability to inhibit distracting or irrelevant information during the numerical Stroop task. Moreover, the HMA model supports the view by ACT that anxiety impairs the efficiency that the task is performed to a greater extent than it impairs the ability to perform the task. The HMA model exerted more effort as shown by longer response times than the LMA model to achieve a similar quality of response accuracy.

The amount of energy (conflict) in the response layer

Procedure. To examine the amount of energy in the response layer when attention is impaired, the model with reduced attention to the numerical size dimension (and not to the physical size dimension) that was consistent with studies of math anxiety was examined further. A congruent trial with stimulus small 1 and large 8 where the LMA and HMA models produced the same simulated response time was chosen and graphed (see Fig 7A). An incongruent trial with stimulus large 1 and small 2 where the HMA model produced a larger simulated response time (by one time step) than the LMA model was chosen and graphed (see Fig 7B). The mean amount of conflict, where conflict is defined as the amount of energy in the response layer, is graphed at each time step. The amount of energy in the response layer was calculated as the product of the activation of the response nodes (see Botvinick et al. [39] for a similar approach).

Results and discussion. Consistent with previous studies [39], incongruent trials experienced more conflict than congruent trials. This is because there is minimal competition during a congruent trial as both the numerical and physical size comparisons activate the same response nodes. However, during an incongruent trial the numerical and physical size comparisons activate competing response nodes, thereby resulting in conflict (that needs to be overcome). Comparison of the LMA model and the HMA model with reduced attention to the numerical stimuli during an incongruent trial showed that the HMA model experienced a weaker activation of the response units which produced less of an opportunity for the existence of conflict. These results suggest that the ability to recognise conflict may be beneficial. Recognising conflict would allow a potential adapting of cognitive control to improve performance. However, as the current simulations did not

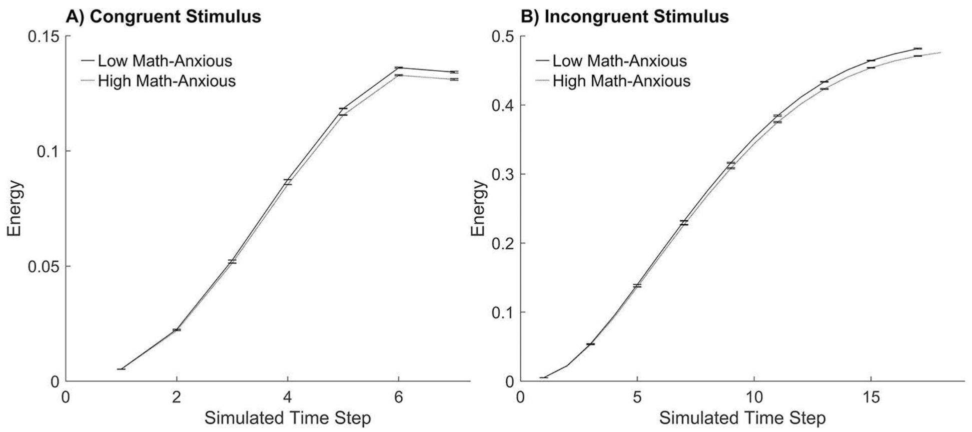

Fig 7. The amount of energy in the response layer during the numerical Stroop task. An example of the mean simulated conflict, which is defined as energy in the response layer (shown on the y-axis), at each simulated time step (shown on the x-axis) across the course of a trial. Each panel shows the results for the low math-anxious model and the high math-anxious model with attention impaired to the (relevant) numerical size dimension. Panel A shows a congruent trial with stimulus small 1 and large 8. Panel B shows an incongruent trial with stimulus large 1 and small 2. Error bars depict the standard error of the mean (the scale of the y-axis differs across the panels to ensure display of error bars).

https://doi.org/10.1371/journal.pone.0295264.g007

have the conflict adaptation module turned on, the simulations did not investigate the effects of conflict adaptation. Therefore, it is unclear as to the potential benefit of recognising the existence of conflict by the LMA model. Furthermore, Bishop [46] found that individuals with high trait anxiety showed less prefrontal cortex activation and slower responses when processing competition than individuals with low trait anxiety in a response-conflict task that required attentional resources. The findings suggested a dysregulation of the recruitment of prefrontal mechanisms required to adjust attentional control when conflict is experienced. Klados et al. [47] used ERP to investigate neural activity in individuals with math anxiety during working memory and arithmetic tasks. They found that individuals with higher levels of self-reported math anxiety showed lower cortical activation at frontocentral and centroparietal locations during the early stages of cognitive processing during simple arithmetic tasks. The results were independent of state and trait anxiety levels. ACT suggests that evidence of weaker neural activity exists if compensatory mechanisms are not employed by anxious individuals when moderate task demands are required [48]. The current simulations did not have the conflict adaptation module turned on, therefore the HMA model did not employ additional compensatory resources, thus resulting in a weaker neural activation in the response layer.

Results and discussion: The symbolic number comparison task

This section describes the neural network model simulations of the symbolic number comparison task, which involves deciding which number is numerically larger when the stimuli are presented in the same physical sizes. First, the LMA model is validated to ensure it simulates various experimental effects. Next, the HMA model is simulated by making parameter changes to the LMA model to assess the effects of these changes and to compare with the results from a group of participants with math anxiety. Below is a description of the simulations of the symbolic number comparison task assessing the behaviour of the model on the distance effect, the size effect, and reduced learning for this task.

Validation of the symbolic number comparison task

Simulation of the symbolic number comparison task. To simulate the symbolic number comparison task, the neural network model that simulated the numerical Stroop task was adapted such that the single-digit comparison module for physical size (used for the irrelevant dimension of the numerical Stroop task) was turned off and the response is generated by comparing numerical sizes. The model simulations use the data set from Dietrich et al. [29] which consists of all combinations of single-digit numbers from 1 to 9 resulting in 72 pairs. The model simulated 30 participants who were low math-anxious. Means for response times on correct trials and error rates were calculated for each simulated participant in each condition.

The simulations that validated the numerical Stroop model included a validation of the symbolic number comparison task, a symbolic number comparison is an inherent requirement of the numerical Stroop task. Consequently, no additional validation of the comparison process of symbolic numbers was required. The model simulations successfully simulated the numerical distance effect and the size effect.

Simulation of changes in the amount of learning. The following simulation demonstrates the effect that a reduction in the training of the connections between the numerical representation and the response has on response times, accuracy, and the distance effect. Previous simulations investigated the effect of a reduction in the training of connection weights between the numerical representations and the response on simulations of the numerical Stroop task. In those simulations the single-digit comparison modules were trained with different values for the number of learning trials, which generated a different set of weights for each training run. The following simulations use these same weights. Model simulations were run for different amounts of training and no other impairments were made to the model (i.e., all models retained 100% attention). The procedure for training the single-digit comparison modules has been described previously.

The results of the simulations on response times, accuracy, and the numerical distance effect were graphed for arbitrary values of the amount of training. In previous simulations when the number of learning trials was 100,000, the models produced 100% accuracy. In the current simulation, the model predicts that as the amount of learning increases, response times will decrease and errors will decrease (see Fig 8). This result is consistent with studies that show learning of basic numerical processing skills across a variety of different tasks improves

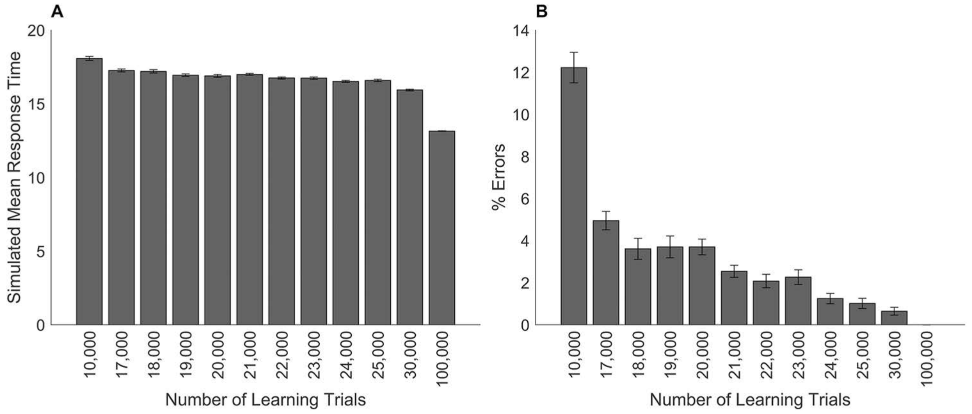

Fig 8. The effect of changing the amount of learning in the symbolic number comparison task. Simulated models' results for different numbers of training trials on performance in the symbolic number comparison task. Panel A shows mean simulated response times. Panel B shows the percentage of errors. Error bars depict the standard error of the mean.

https://doi.org/10.1371/journal.pone.0295264.g008

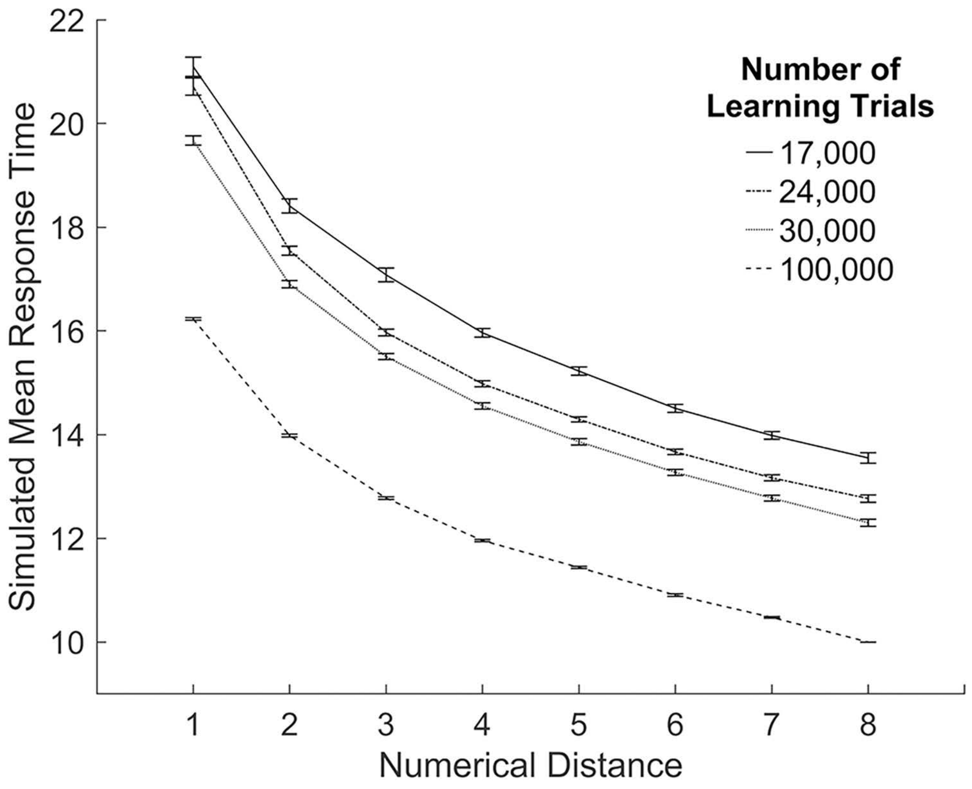

Fig 9. The numerical distance effect across learning in the symbolic number comparison task. The numerical distance effect is shown for different values of learning trials. The x-axis depicts the distance between the numerical stimuli. Error bars depict the standard error of the mean.

https://doi.org/10.1371/journal.pone.0295264.g009

response times and accuracy (e.g., Landerl [44]). Fig 9 shows the results for the numerical distance effect where overall response times decrease for each numerical distance as learning increases. This result is consistent with studies showing changes in the numerical distance effect for symbolic number comparison during development (e.g., Landerl and Kölle [45]).

Math anxiety experimental results for the symbolic number comparison task

The symbolic number comparison task has been studied within the math anxiety literature with mixed results. Some studies have found differences between individuals high and low in math anxiety in overall response times, for distance and size effects, and some have not. Most studies have found that there are no differences in error rates between individuals high and low in math anxiety.

Procedure for the simulation of HMA models

The model simulations use the same data set and conditions that were used to validate the symbolic number comparison model in the previous section. There were 30 simulated participants for both the LMA and the HMA models. As in the numerical Stroop model, the connection weights between the input layer and the comparison layer of the single-digit comparison module for numerical size were different for each simulated participant within each LMA or HMA model, and were the same for each matched LMA and HMA participant. This facilitated

a comparison of the results of the LMA and HMA models. Means for response times on correct trials and error rates were calculated for each simulated participant in each condition.

Simulation of the HMA model with impaired attention

Procedure. This simulation investigates the effect of reduced attention during performance of the symbolic number comparison task. The numerical size single-digit comparison modules were trained to 100% accuracy in the initial simulations of the numerical Stroop task. The errors in those simulations resulted from the conflict between the relevant and irrelevant dimensions. The first simulation here retains these same connection weights between the input layer and the comparison layer resulting in 100% accuracy for the single-digit comparison task. The results from the numerical Stroop model simulations proposed that impairing attention to the numerical size dimension qualitatively replicated results from experimental studies on math anxiety. Therefore, for this simulation attention was impaired to the HMA model on the numerical size dimension. This was achieved by reducing the activation of the neurons in the task demand units for the numerical size to 95% as in the numerical Stroop model simulations.

Results. Fig 10 shows the results for the math anxiety experimental data of Dietrich et al. [29] and the model simulations. These authors reported results for low, middle, and high math anxiety as assessed by the Abbreviated Math Anxiety Scale (AMAS; [49]). They did not find overall differences in response times between the LMA and HMA groups. However, they found a more pronounced distance effect in response times for the HMA group than the LMA group. The simulation results show that the HMA model produced longer response times than the LMA model. The more attention was reduced, the longer the response time. The LMA model and the HMA model produced reliable numerical distance effects where it is faster to compare two numbers and decide which is the largest when the distance between the numbers is large than when the distance between the numbers is small. The size of the distance effect for the HMA model was similar to the distance effect in the LMA model. As the

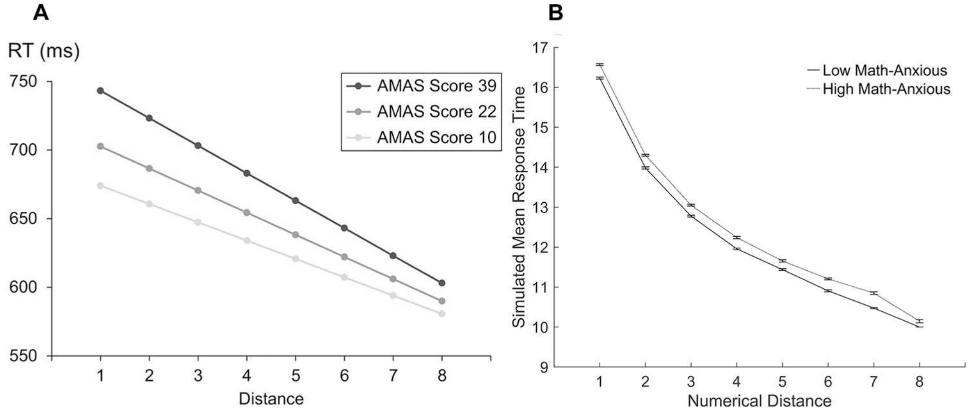

Fig 10. Model simulations of the symbolic number comparison task with attention impaired. Response times for the distance effect in symbolic number comparison, as a function of math anxiety, are shown for experimental and modelling results. (A) Experimental results of the estimated distance effects for participants with low math anxiety (Abbreviated Math Anxiety Scale (AMAS) score = 10), middle math anxiety (AMAS score = 22), and high math anxiety (AMAS score = 39). Reprinted from "The Influence of Math Anxiety on Symbolic and Non-Symbolic Magnitude Processing" by Dietrich JF, Huber S, Moeller K, Klein E. Frontiers in Psychology. 2015;6(1621). p. 6. doi: 10.3389/fpsyg.2015.01621. CC BY 4.0. [29] (B) Results of the high math-anxious model simulation with attention to the numerical sizes impaired. The y-axis shows the simulated mean response time. The x-axis shows the distance between the numerical stimuli. Error bars depict the standard error of the mean.

https://doi.org/10.1371/journal.pone.0295264.g010

single-digit comparison module was trained to 100% accuracy, there were no differences in error rates between the LMA and the HMA model.

Discussion. The model simulated the effect of reducing attentional control to numerical stimuli by reducing the activation of the corresponding task demand units, which resulted in longer response times. Studies on math anxiety have found that performance is affected more on tasks that require more working memory resources, as these resources are specifically disrupted by anxiety [50]. Recent research involving neuroimaging and ERP during numerical tasks have shown that cognitive processing differs between individuals with and without math anxiety, even though they may achieve similar performance outcomes (see [51]). Furthermore, Batashvili et al. [52] found that individuals with math anxiety experienced a threat-related response just by observing simple numerical stimuli. Therefore, the model of reduced attention to numerical stimuli is consistent with the findings that math anxiety may reduce attention to numerical stimuli, even on basic numerical tasks if there is sufficient anxiety.

However, there are limitations of the simulations of reduced attention to numerical stimuli as a model of math anxiety. The LMA and HMA models did not produce any errors as the single-digit numerical size comparison module had been trained to successfully compare the numbers and decide which was numerically largest. Even though error rates are extremely low for the symbolic comparison task and that generally there are no differences between the LMA and HMA groups in the literature for accuracy, this model simulation did not account for error rates. Further, in some studies individuals with and without math anxiety show similar performance outcomes for response times and accuracy, yet they have shown differences in the processing of numerical stimuli as demonstrated by ERP measures (e.g., Pletzer et al. [53]). The current simulations did not account for the condition where the LMA and HMA models produced similar performance outcomes for response times. However, the model was not designed to model all aspects of working memory and it is not a biologically plausible model. Instead, its aim was to identify the underlying cognitive factors associated with math anxiety, and the modelling supports the view that math anxiety affects attentional processes.

Simulation of the HMA model with reduced learning

Procedure. Dietrich et al. [29] examined the effects of math anxiety on the symbolic number comparison task and suggested that individuals with math anxiety may have less trained connections between the numerical representations and the response. These simulations test the effect of reduced learning with and without reduced attentional control to the numerical size dimension during performance of the symbolic number comparison task. To examine the effect of reduced learning, an LMA model and an HMA model were chosen initially with a specific amount of learning trials such that the error rates for these models were close to the results of experimental studies and were consistent with experimental findings where there were no differences between them. A Mann-Whitney equivalent test was performed on the percentage of errors in the LMA and HMA models to confirm there were no significant differences ($U = 888.5$, $n_1 = n_2 = 30$, $p = .696$) as the data was not normally distributed. Dietrich et al. [29] reported an overall error rate for symbolic number comparison of 3.82%. The LMA model was chosen with 20,000 learning trials and 3.7% errors. The HMA model was chosen with 18,000 learning trials and 3.61% errors. These values were chosen so that there were no differences in the percentage of errors between the LMA and HMA models, yet the number of learning trials were far enough apart for there to be a difference between the models in response times. Subsequently, the chosen LMA and HMA models were simulated with and without reduced attention. To simulate reduced attention, the numerical

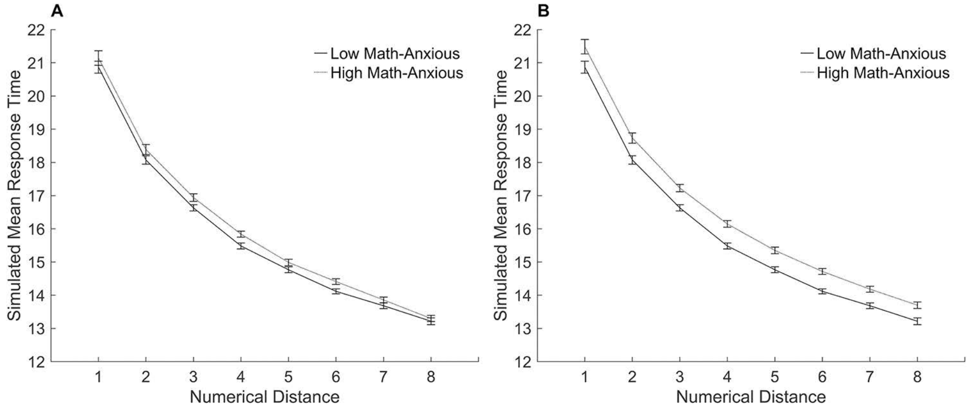

Fig 11. Model simulations of the symbolic number comparison task with reduced learning. Mean simulated response times for the numerical distance effect for the low and high math-anxious models. (A) The low math-anxious model has 20,000 learning trials, the high math-anxious model has 18,000 learning trials. (B) The low math-anxious model has 20,000 learning trials, the high math-anxious model has 18,000 learning trials and attention reduced to the numerical stimuli. The y-axis shows the simulated mean response time. The x-axis shows the distance between the numbers. Error bars depict the standard error of the mean.

https://doi.org/10.1371/journal.pone.0295264.g011

size dimension of the HMA model was reduced to 95%. This impairment was chosen because it produced a model of math anxiety in previous simulations.

Results. Fig 11 shows the results of the simulations with reduced learning, with and without reduced attentional control.

Reduced learning without attention impaired. The results of the model simulations of reduced training trials on response times when there is no impairment in attention are presented. From Fig 11A you can see that there are no differences in the response times as a result of the reduction in the number of training trials in the HMA model results. The LMA model and the HMA model produced reliable numerical distance effects. The size of the distance effect for the HMA model was similar to the distance effect in the LMA model.

Reduced learning with reduced attention. The HMA model was simulated with reduced training trials and attention reduced to the numerical size dimension. The HMA model produced longer overall response times than the LMA model (Fig 11B). The HMA model did not differ from the LMA model on the percentage of errors. Both models produced a reliable numerical distance effect. The size of the distance effect for the HMA model was similar to the distance effect in the LMA model. The size effect was examined for the combination of stimulus pairs "1 2" (which includes "2 1') and "8 9" (which includes "9 8") as they were as extreme as possible to compare, as in the study by Núñez-Peña and Suárez-Pellicioni [28] who found marginal differences between the LMA and HMA groups for the size effect. As in this study, an interference effect was calculated for the size effect where the response times for small numbers was subtracted from large numbers. Both models produced a reliable size effect. The interference effect for the size effect of the HMA model was similar to the interference effect in the LMA model.

Discussion. After simulating the models with reduced learning, the accuracy of trials was reduced and only the HMA model with reduced learning and reduced attention produced longer response times than the LMA model, supporting the disruption account where if individuals with math anxiety have less trained connections between the numerical representation and the response, math anxiety is characterised by a disruption to attention that leads to poor performance on numerical tasks. The HMA model with reduced learning and reduced attention to numerical stimuli simulated the distance effect and the size effect,

as this model produced the best fit to empirical data. Núñez-Peña and Suárez-Pellicioni [28] found marginal differences between the LMA and HMA groups for the distance and size effect. However, some subsequent findings in the research literature show no differences between groups suggesting that the marginal differences between LMA and HMA groups may be unreliable. In the current modelling, the HMA model did not produce more pronounced distance or size effects than the LMA model. Dietrich et al.'s [29] suggestion that individuals with math anxiety may have less trained connections was motivated by the fact that in some experimental studies individuals high in math anxiety had more pronounced distance effects than individuals low in math anxiety, and the distance effect in symbolic number comparison indexes comparison processes between the numerical representation and the response [23–25]. Furthermore, Colomé [30] suggested that the differences in the behavioural studies may also be due to either experimental design or that motivation and attitudes towards mathematics were not controlled for in the studies and could explain the variability between them. The results of the current modelling do not predict that reduced attention due to math anxiety affects the distance effect and the size effect. These findings are consistent with the results of behavioural studies where more pronounced distance and size effects in individuals with math anxiety is not a robust finding. However, there are limitations to this conclusion. The models simulating reduced learning were chosen by matching error rates from experimental studies. This resulted in a small difference between the amount of learning performed by the LMA and HMA models, which may have influenced the results. Alternatively, another interpretation of the results of the current modelling is that there may be other factors related to working memory or attentional control that the model does not account for that could produce more pronounced effects.

Conclusions

The aim of the current study was to investigate using neural network modelling whether poor performance due to math anxiety is due to impaired attentional and inhibitory functions, as suggested by the disruption account. The claims by ACT on the effect of anxiety on task performance were used as a framework to further explain these impairments and investigate their application to math anxiety. To our knowledge, this is the first study to simulate math anxiety using neural network modelling. Our neural network model has provided a means to test theories on math anxiety and attentional control. Two numerical tasks, the numerical Stroop and symbolic number comparison, were modelled on one neural network model architecture. The numerical Stroop task was modelled as it specifically requires attention to inhibit irrelevant information. Modelling symbolic number comparison allowed investigation of the role of attention during a basic numerical task as results of studies are inconsistent and it has been suggested that individuals high and low in math anxiety may differ in attentional control. Furthermore, as Dietrich et al. [29] suggested that the larger distance effects in math anxious individuals may be due to less trained connections between the numerical representation and the response in deciding which number is larger (as the comparison distance effect indexes comparison processes), we investigated the effect of reducing the amount of learning in the model, with and without attention reduced. Initially, the model was validated to ensure it simulated the various empirical effects for both tasks. Subsequently, similar modifications were made to the network for both tasks to simulate different cognitive conditions. The results of the simulations were then compared to experimental results on math anxiety to decide whether they were a suitable model of math anxiety. The modifications resulted in similar conclusions for both tasks, that math anxiety is associated with reduced attentional control to numerical stimuli. Overall, the results support the disruption account as it relates to ACT that math anxiety disrupts attention and inhibitory functions that result in underperformance in mathematical tasks [5, 6].

Both tasks were firstly simulated with impaired attention to the numerical stimuli by reducing the activation of the task demand units representing the top-down goal-directed attention system. The models both showed that reduced attention to numerical stimuli resulted in longer response times and no changes in accuracy. The numerical Stroop task additionally involves inhibiting attention to the irrelevant physical size dimension. When the modelling involved reducing attention to the numerical size (relevant) dimension it produced results qualitatively similar to those of experimental studies on mathematics anxiety. However, when attention was only reduced to the physical size dimension it did not produce results that matched those of experimental studies on mathematics anxiety. These findings are in support of the ACT which claims that anxiety increases the influence of the stimulus-driven attentional system and decreases the influence of the goal-directed attentional system affecting the ability to inhibit distracting or irrelevant information for the current task.

Next, the symbolic number comparison task was simulated with reduced learning with and without reduced attention to the numerical stimuli. Reduced learning was simulated by changing the parameter for the number of learning trials, which increased response times and error rates on both tasks. The models with reduced learning and no impairments of attention did not produce a good qualitative fit to empirical data. However, the models with reduced learning and reduced attention to the numerical stimuli provided the best qualitative fit to previous studies in math anxiety and resulted in impaired performance. These results support the view that if individuals have less trained connections possibly due to the avoidance of numerical tasks, that math anxiety is further characterised by a disruption to the attention to numerical stimuli during processing and recall.

Future work

Carey et al. [5] describes that math anxiety can impact learning due to avoidance and subsequently impact processing and recall because the anxiety disrupts working memory resources (the disruption account). The model simulations did not investigate the effect of anxiety during learning. This was outside the scope of the current study. Future work could involve impairing attention to the models during training.

In the numerical Stroop task, future work should examine conflict adaptation effects due to reduced attentional control from math anxiety. In conclusion, the models predict that reduced attention to the numerical stimulus dimension, and therefore to the top-down goal-directed attentional system, due to math anxiety reduces the amount of conflict (i.e., energy in the response layer) which may affect the ability to be able to adapt to the presence of conflict during processing. Suárez-Pellicioni, Núñez-Peña and Colomé [12] examined conflict adaptation effects and found that math anxiety was associated with a compensatory recruitment of cognitive control after experiencing conflicting information.

Supporting information

S1 File. Model architecture: Further details and equations.
(PDF)

S2 File. Simulation of the speed-accuracy trade-off during performance of the numerical Stroop task.
(PDF)

Acknowledgments

This article is part of the first author's Master of Research thesis. We thank Charlotte S. Barber for help with designing figures.

Author contributions

Conceptualization: Angela C. Rose, Hany Alashwal, Ahmed A. Moustafa.

Formal analysis: Angela C. Rose, Gabrielle Weidemann.

Investigation: Angela C. Rose.

Methodology: Angela C. Rose, Ahmed A. Moustafa.

Project administration: Gabrielle Weidemann.

Software: Angela C. Rose, Gabrielle Weidemann.

Supervision: Ahmed A. Moustafa, Gabrielle Weidemann.

Writing – original draft: Angela C. Rose, Gabrielle Weidemann.

Writing – review & editing: Angela C. Rose, Hany Alashwal, Gabrielle Weidemann.

References

1. Ashcraft MH, Faust MW. Mathematics anxiety and mental arithmetic performance: An exploratory investigation. Cognition & Emotion. 1994;8(2):97–125. https://doi.org/10.1080/02699939408408931
2. Organisation for Economic Co-operation and Development. PISA 2012 results: Ready to learn: Students' engagement, drive and self-beliefs (Volume III): Vol. III. OECD Publishing; 2013. https://doi.org/10.1787/9789264201170-en
3. Lyons IM, Beilock SL. Mathematics anxiety: Separating the math from the anxiety. Cerebral Cortex. 2012;22(9):2102–10. https://doi.org/10.1093/cercor/bhr289 PMID: 22016480
4. Office of the Chief Scientist. Transforming STEM teaching in Australian primary schools: Everybody's business. 2015. Available from: https://www.chiefscientist.gov.au/sites/default/files/Transforming-STEM-teaching_FINAL.pdf
5. Carey E, Hill F, Devine A, Szűcs D. The chicken or the egg? The direction of the relationship between mathematics anxiety and mathematics performance. Frontiers in Psychology. 2016;6:1987. https://doi.org/10.3389/fpsyg.2015.01987 PMID: 26779093
6. Ramirez G, Shaw ST, Maloney EA. Math anxiety: Past research, promising interventions, and a new interpretation framework. Educational Psychologist. 2018;53(3):145–64. https://doi.org/10.1080/00461520.2018.1447384
7. Derakshan N, Eysenck MW. Anxiety, processing efficiency, and cognitive performance: New developments from attentional control theory. European Psychologist. 2009;14(2):168–76. https://doi.org/10.1027/1016-9040.14.2.168
8. Eysenck MW, Derakshan N, Santos R, Calvo MG. Anxiety and cognitive performance: Attentional control theory. Emotion. 2007;7(2):336–53. https://doi.org/10.1037/1528-3542.7.2.336 PMID: 17516812
9. Corbetta M, Shulman GL. Control of goal-directed and stimulus-driven attention in the brain. Nat Rev Neurosci. 2002;3(3):201–15. https://doi.org/10.1038/nrn755 PMID: 11994752
10. Stroop JR. Studies of interference in serial verbal reactions. Journal of Experimental Psychology. 1935;18(6):643–62. https://doi.org/10.1037/h0054651
11. Hopko DR, McNeil DW, Gleason PJ, Rabalais AE. The emotional Stroop paradigm: Performance as a function of stimulus properties and self-reported mathematics anxiety. Cognitive Therapy and Research. 2002;26(2):157–66. https://doi.org/10.1023/A:1014578218041.
12. Suárez-Pellicioni M, Núñez-Peña MI, Colomé À. Reactive recruitment of attentional control in math anxiety: An ERP study of numeric conflict monitoring and adaptation. PLoS ONE. 2014;9(6):e99579. https://doi.org/10.1371/journal.pone.0099579 PMID: 24918584
13. Kalanthroff E, Henik A, Derakshan N, Usher M. Anxiety, emotional distraction, and attentional control in the Stroop task. Emotion. 2016;16(3):293–300. https://doi.org/10.1037/emo0000129 PMID: 26571078
14. Richards A, Richards LC, McGeeney A. Anxiety-related Stroop interference in adolescents. The Journal of General Psychology. 2000;127(3):327–33. https://doi.org/10.1080/00221300009598587 PMID: 10975428
15. Justicia-Galiano M-J, Pelegrina S, Lechuga M, Gutiérrez-Palma N, Martín-Puga E-M, Lendínez C. Math anxiety and its relationship to inhibitory abilities and perceived emotional intelligence. Anales de Psicología. 2016;32(1):125–31. https://doi.org/10.6018/analesps.32.1.194891

16. Mammarella IC, Caviola S, Giofrè D, Borella E. Separating math from anxiety: The role of inhibitory mechanisms. Applied Neuropsychology: Child. 2018;7(4):342–53. https://doi.org/10.1080/21622965.2017.1341836 PMID: 28682117

17. Ashkenazi S. Intentional and automatic processing of numerical information in mathematical anxiety: Testing the influence of emotional priming. Cognition and Emotion. 2018;32(8):1700–7. https://doi.org/10.1080/02699931.2018.1435504 PMID: 29400601

18. Hartwright CE, Looi CY, Sella F, Inuggi A, Santos FH, González-Salinas C, et al. The neurocognitive architecture of individual differences in math anxiety in typical children. Scientific Reports. 2018;8(1):8500. https://doi.org/10.1038/s41598-018-26912-5 PMID: 29855608

19. Liu J, Li J, Peng W, Feng M, Luo Y. EEG correlates of math anxiety during arithmetic problem solving: Implication for attention deficits. Neuroscience Letters. 2019;703:191–7. https://doi.org/10.1016/j.neulet.2019.03.047 PMID: 30928479

20. Pizzie RG, Kraemer DJM. Avoiding math on a rapid timescale: Emotional responsivity and anxious attention in math anxiety. Brain and Cognition. 2017;118:100–7. https://doi.org/10.1016/j.bandc.2017.08.004 PMID: 28826050

21. Dehaene S. Précis of the number sense. Mind & Language. 2001;16(1):16–36. https://doi.org/10.1111/1468-0017.00154

22. Moyer RS, Landauer TK. Time required for judgements of numerical inequality. Nature. 1967;215:1519–20. https://doi.org/10.1038/2151519a0 PMID: 6052760

23. van Opstal F, Gevers W, de Moor W, Verguts T. Dissecting the symbolic distance effect: Comparison and priming effects in numerical and nonnumerical orders. Psychonomic Bulletin & Review. 2008;15(2):419–25. https://doi.org/10.3758/pbr.15.2.419 PMID: 18488662

24. van Opstal F, Verguts T. The origins of the numerical distance effect: The same–different task. Journal of Cognitive Psychology. 2011;23(1):112–20. https://doi.org/10.1080/20445911.2011.466796

25. Verguts T, Fias W, Stevens M. A model of exact small-number representation. Psychonomic Bulletin and Review. 2005;12(1):66–80. https://doi.org/10.3758/bf03196349 PMID: 15945201

26. Feigenson L, Dehaene S, Spelke E. Core systems of number. Trends in Cognitive Sciences. 2004;8(7):307–14. https://doi.org/10.1016/j.tics.2004.05.002 PMID: 15242690

27. Maloney EA, Ansari D, Fugelsang JA. The effect of mathematics anxiety on the processing of numerical magnitude. Quarterly Journal of Experimental Psychology. 2011;64(1):10–6. https://doi.org/10.1080/17470218.2010.533278 PMID: 21113858

28. Núñez-Peña MI, Suárez-Pellicioni M. Less precise representation of numerical magnitude in high math-anxious individuals: An ERP study of the size and distance effects. Biological Psychology. 2014;103:176–83. https://doi.org/10.1016/j.biopsycho.2014.09.004 PMID: 25224181

29. Dietrich JF, Huber S, Moeller K, Klein E. The influence of math anxiety on symbolic and non-symbolic magnitude processing. Frontiers in Psychology. 2015;6:1621. https://doi.org/10.3389/fpsyg.2015.01621 PMID: 26579012

30. Colomé À. Representation of numerical magnitude in math-anxious individuals. Quarterly Journal of Experimental Psychology. 2019;72(3):424–35. https://doi.org/10.1177/1747021817752094 PMID: 29359641

31. Ashcraft MH, Krause JA, Hopko DR. Is math anxiety a mathematical learning disability? In Berch DB, Mazzocco MMM, editors. Why is math so hard for some children? The nature and origins of mathematical learning difficulties and disabilities. Paul H. Brookes Publishing Co.; 2007. pp. 329–48.

32. Moeller K, Huber S, Nuerk H-C, Willmes K. Two-digit number processing: Holistic, decomposed or hybrid? A computational modelling approach. Psychological Research. 2011;75(4):290–306. https://doi.org/10.1007/s00426-010-0307-2 PMID: 20798955

33. Huber S, Moeller K, Nuerk H-C, Macizo P, Herrera A, Willmes K. Cognitive control in number processing-a computational model. In West R, Stewart T, editors. Proceedings of the 12th International Conference on Cognitive Modeling. Ottawa, Canada: Carleton University; 2013. pp. 185–90. https://doi.org/10.13140/2.1.4137.4729

34. Verguts T, Notebaert W. Hebbian learning of cognitive control: Dealing with specific and nonspecific adaptation. Psychological Review. 2008;115(2):518–25. https://doi.org/10.1037/0033-295X.115.2.518 PMID: 18426302

35. Huber S, Nuerk H-C, Willmes K, Moeller K. A General Model Framework for Multisymbol Number Comparison. Psychological Review. 2016;123(6):667–95. https://doi.org/10.1037/rev0000040 PMID: 27617347

36. Huber S, Moeller K, Nuerk H-C, Willmes K. A computational modeling approach on three-digit number processing. Topics in Cognitive Science. 2013;5(2):317–34. https://doi.org/10.1111/tops.12016 PMID: 23460505
37. Huber S, Klein E, Willmes K, Nuerk H-C, Moeller K. Decimal fraction representations are not distinct from natural number representations—evidence from a combined eye-tracking and computational modeling approach. Frontiers in Human Neuroscience. 2014;8:172. https://doi.org/10.3389/fnhum.2014.00172 PMID: 24744717
38. Santens S, Verguts T. The size congruity effect: Is bigger always more? Cognition. 2011;118(1):94–110. https://doi.org/10.1016/j.cognition.2010.10.014 PMID: 21074146
39. Botvinick MM, Braver TS, Barch DM, Carter CS, Cohen JD. Conflict monitoring and cognitive control. Psychological Review. 2001;108(3):624–52. https://doi.org/10.1037/0033-295x.108.3.624 PMID: 11488380
40. Schmidt JR. Evidence against conflict monitoring and adaptation: An updated review. Psychonomic Bulletin & Review. 2019;26(3):753–71. https://doi.org/10.3758/s13423-018-1520-z PMID: 30511233
41. Moustafa AA, Porter A, Megreya AM. Mathematics anxiety and cognition: An integrated neural network model. Rev. Neurosci. 2020;31(3):287–96. https://doi.org/10.1515/revneuro-2019-0068 PMID: 31730536
42. Verguts T, Fias W. Lexical and syntactic structures in a connectionist model of reading multi-digit numbers. Connection Science. 2006;18(3):265–83. https://doi.org/10.1080/09540090600639396
43. Cohen JD, Dunbar K, McClelland JL. On the control of automatic processes: A parallel distributed processing account of the Stroop effect. Psychological Review. 1990;97(3):332–61. https://doi.org/10.1037/0033-295x.97.3.332 PMID: 2200075
44. Landerl K. Development of numerical processing in children with typical and dyscalculic arithmetic skills-a longitudinal study. Frontiers in Psychology. 2013;4:459. https://doi.org/10.3389/fpsyg.2013.00459 PMID: 23898310
45. Landerl K, Kölle C. Typical and atypical development of basic numerical skills in elementary school. Journal of Experimental Child Psychology. 2009;103(4):546–65. https://doi.org/10.1016/j.jecp.2008.12.006 PMID: 19254797
46. Bishop SJ. Trait anxiety and impoverished prefrontal control of attention. Nature Neuroscience. 2009;12(1):92–9. https://doi.org/10.1038/nn.2242 PMID: 19079249
47. Klados MA, Simos P, Micheloyannis S, Margulies D, Bamidis PD. ERP measures of math anxiety: How math anxiety affects working memory and mental calculation tasks? Frontiers in Behavioral Neuroscience. 2015;9:282. https://doi.org/10.3389/fnbeh.2015.00282 PMID: 26578912
48. Berggren N, Derakshan N. Attentional control deficits in trait anxiety: Why you see them and why you don't. Biological Psychology. 2013;92(3):440–6. https://doi.org/10.1016/j.biopsycho.2012.03.007 PMID: 22465045
49. Hopko DR, Mahadevan R, Bare RL, Hunt MK. The Abbreviated Math Anxiety Scale (AMAS): Construction, validity, and reliability. Assessment. 2003;10(2):178–82. https://doi.org/10.1177/1073191103010002008 PMID: 12801189
50. Faust MW, Ashcraft MH, Fleck DE. Mathematics anxiety effects in simple and complex addition. Mathematical Cognition. 1996;2(1):25–62. https://doi.org/10.1080/135467996387534
51. Artemenko C, Daroczy G, Nuerk H-C. Neural correlates of math anxiety–an overview and implications. Frontiers in Psychology. 2015;6:1333. https://doi.org/10.3389/fpsyg.2015.01333 PMID: 26388824
52. Batashvili M, Staples P, Baker IS, Sheffield D. The neurophysiological relationship between number anxiety and the EEG gamma-band. Journal of Cognitive Psychology. 2020;32(5–6):580–5. https://doi.org/10.1080/20445911.2020.1778006
53. Pletzer B, Kronbichler M, Nuerk H-C, Kerschbaum HH. Mathematics anxiety reduces default mode network deactivation in response to numerical tasks. Frontiers in Human Neuroscience. 2015;9:202. https://doi.org/10.3389/fnhum.2015.00202 PMID: 25954179
